# Perovskite Solar Cells for Extreme Environments: Tailoring Material Design and Exploring New Opportunities

**DOI:** 10.1007/s40820-026-02173-0

**Published:** 2026-04-13

**Authors:** Wooyeon Kim, Bonkee Koo, Min Jae Ko

**Affiliations:** 1 Department of Chemical Engineering, Hanyang University, Seoul, 04763 Republic of Korea; 2 Clean-Energy Research Institute, Hanyang University, Seoul, 04763 Republic of Korea

**Keywords:** Perovskite solar cells, Material design, Extreme environments, Space, Underwater, Durability

## Abstract

A stable and independent power supply is essential in extreme environments, where access to power infrastructure or opportunities for resupply are limited. Conventional batteries and fuel-powered generators are often heavy and require specialized maintenance. In contrast, solar cells can generate electricity directly from sunlight without reliance on external fuel sources. Among next-generation photovoltaic technologies, perovskite solar cells (PSCs) offer notable advantages, including tunable bandgaps and a high absorption coefficient, which enable spectral matching and stable operation even under low-irradiance conditions. Furthermore, PSCs are thin, lightweight, and mechanically flexible, allowing integration into mobile electronics and aerial systems such as drones and satellites. Despite these advantages, PSCs were previously considered unsuitable for deployment in harsh environments due to the intrinsic instability of perovskite absorbers, which are prone to degradation under external stressors such as heat, humidity, and continuous radiation. Recent progress in interface passivation, encapsulation, and the development of self-healing perovskite materials has significantly enhanced the long-term stability of PSCs, thereby enabling their potential application in extreme environmental conditions. In this review, we categorize the environmental threats relevant to space, underwater, desert, and polar regions, and analyze the specific stressors associated with each setting. We then examine recent technological advances that address these stressors and evaluate the practical feasibility of PSC operation under such conditions. The review concludes by identifying remaining challenges and offering perspectives on the future implementation of PSCs in extreme environments.

## Highlights

- This review discusses the deployment of perovskite solar cells (PSCs) in extreme environments such as space, underwater, desert, and polar regions.
- We examine recent strategies to enhance the long-term stability of PSCs through defect passivation, interface engineering, encapsulation, and self-healing perovskites, with respect to degradation mechanisms under continuous irradiation, elevated temperatures, and high humidity.
- Guidelines are also proposed for future stability testing and practical performance criteria that PSCs must satisfy for reliable operation under extreme environmental conditions.

## Graphical abstract

**Figure:**
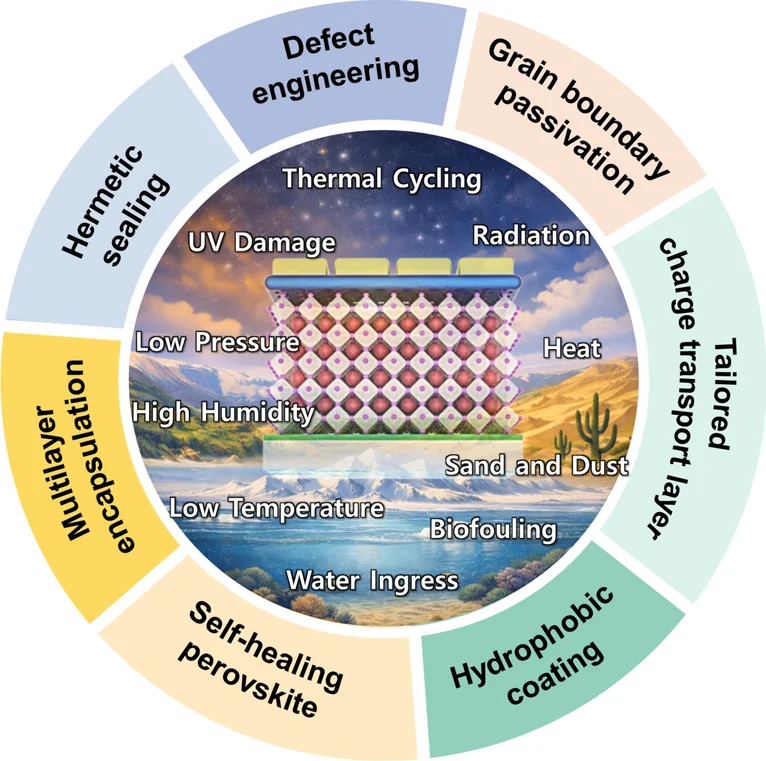

## Introduction

As human activity expands into unexplored regions, the need for a stable and continuous local power supply becomes increasingly critical [1–3]. However, power-generation infrastructure remains limited in extreme environments such as outer space, underwater, deserts, polar regions, and high-altitude areas. In these settings, exploration equipment, communication modules, and sensors must operate continuously, necessitating an independent and reliable power source [4, 5]. To date, batteries and fossil-fuel-based systems have served as the primary energy sources in such environments. However, these systems are heavy, mechanically complex, and difficult to transport or maintain, posing logistical challenges and raising safety concerns. Consequently, the development of next-generation power-generation technologies that are lightweight, structurally simple, and capable of long-term operation without external fuel is essential to ensure a sustainable energy supply under harsh conditions.

Among current power-generation options, solar cells represent the most efficient approach for self-sustaining electricity production, directly converting sunlight into electrical energy without the need for fuel. They offer advantages in terms of low weight, structural simplicity, and reduced maintenance compared to conventional systems. In space applications, multijunction III–V compound solar cells serve as established benchmarks for photovoltaic performance owing to their radiation tolerance and high power-conversion efficiency (PCE) [6]. In terrestrial applications, silicon (Si) solar cells remain dominant owing to their cost-effectiveness and long-term operational stability [7]. For applications requiring mechanical compliance, thin-film photovoltaics offer clear advantages in lightweight and flexible form factors [8, 9]. In particular, CIGS- and organic photovoltaic (OPV)-based devices have been actively explored for wearable electronics and aerial platforms. Despite these environment-specific strengths, the applicability of incumbent photovoltaic technologies remains limited under extreme environmental conditions. III–V solar cells rely on costly materials and complex fabrication processes [10]. By contrast, Si solar cells are rigid and heavy, which limits their applicability in mobile or weight-sensitive applications. CIGS and CdTe solar cells suffer from photothermal degradation, whereas organic photovoltaic and dye-sensitized solar cells (DSSCs) undergo chemical degradation under combined stress from heat, light, and oxygen [8]. These limitations reduce the environmental compatibility of conventional photovoltaic technologies, motivating the exploration of photovoltaic technologies better suited for extreme-environment deployment.

**Fig. 1:**
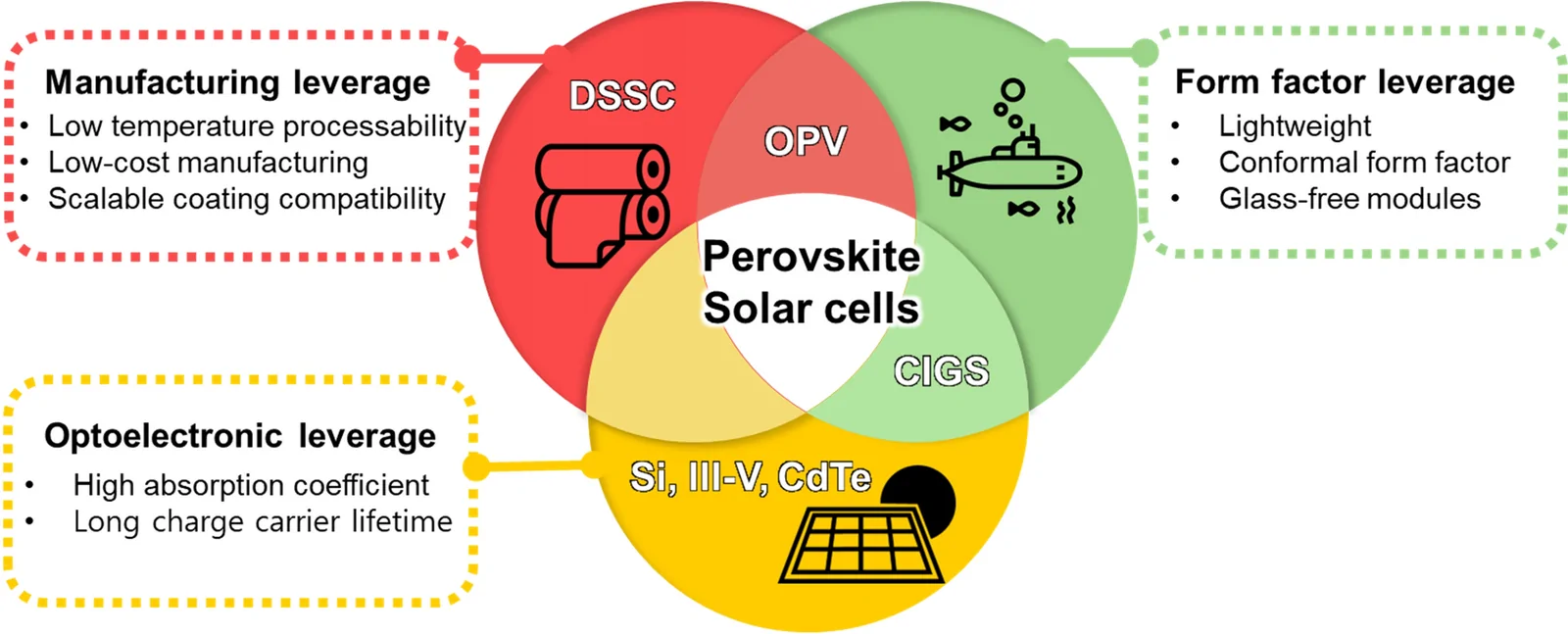
Venn diagram comparing perovskite solar cells (PSCs) with representative photovoltaic technologies for deployment under extreme environmental conditions. The three domains represent optoelectronic, manufacturing, and form-factor leverage

To address these limitations, perovskite solar cells (PSCs) offer a promising pathway toward next-generation photovoltaics with enhanced environmental adaptability. Figure 1 summarizes why PSCs satisfy the combined optoelectronic, manufacturing, and form-factor requirements for deployment under extreme environmental conditions, compared to incumbent photovoltaic technologies. Halide perovskites enable precise bandgap tunability (1.2–2.3 eV) through compositional engineering, allowing spectral matching across a wide range of illumination conditions [9, 10]. In addition, their high absorption coefficients (> 10^5^ cm^−1^) facilitate efficient light harvesting in sub-100 nm layers, which is advantageous for lightweight device design and operation under low-irradiance conditions [11, 12]. PSCs can be manufactured via low-temperature solution processes without the need for vacuum deposition or complex synthesis, compatible with scalable manufacturing approaches. Their compatibility with flexible substrates allows integration into mobile systems such as drones and wearable electronics.

Although early PSCs were highly susceptible to degradation under thermal stress, moisture, and ultraviolet (UV) radiation [13–15], various stabilization strategies have since been developed. These include surface and interface passivation, two-dimensional (2D)/three-dimensional (3D) perovskite heterostructure engineering, advanced encapsulation methods, the incorporation of self-healing perovskite materials, and the implementation of charge-transport layers as protective interlayers [16–20]. As a result, modern PSCs exhibit significantly improved radiation hardness, moisture resistance, and in some cases, self-recovery behavior, enabling sustained photovoltaic performance under extreme environmental conditions.

Several stabilization strategies developed for terrestrial PSCs, such as defect passivation and conventional encapsulation, remain applicable to extreme environments because they address intrinsic material instability. However, extreme environments introduce additional stress factors, including high-energy radiation, low pressure, prolonged thermal cycling, and continuous water exposure, which are not sufficiently addressed by standard terrestrial approaches. Therefore, reliable operation under these conditions requires environment-specific design modifications, including radiation-tolerant charge-transport layers, hermetic encapsulation with resistance to degassing, and stress adaptive or self-healing material systems.

From a market perspective, photovoltaic applications in extreme environments are highly diverse and application specific, rather than forming a single large-scale market. Consequently, performance requirements vary substantially depending on the intended use case. Recent studies indicate that, in such niche applications, system-level factors such as weight and ease of integration often outweigh module-level cost metrics, particularly for space, mobile, and off-grid systems [21, 22]. Within this context, PSCs are not expected to serve as universal replacements for established photovoltaic technologies. Rather, they are suited to addressing specific application gaps. These gaps arise when achieving optoelectronic design flexibility, scalable manufacturing, and lightweight device configurations simultaneously. Accordingly, module design and overall system architecture should be tailored to the requirements of each application. Performance metrics should then be selected based on the operating environment.

**Fig. 2:**
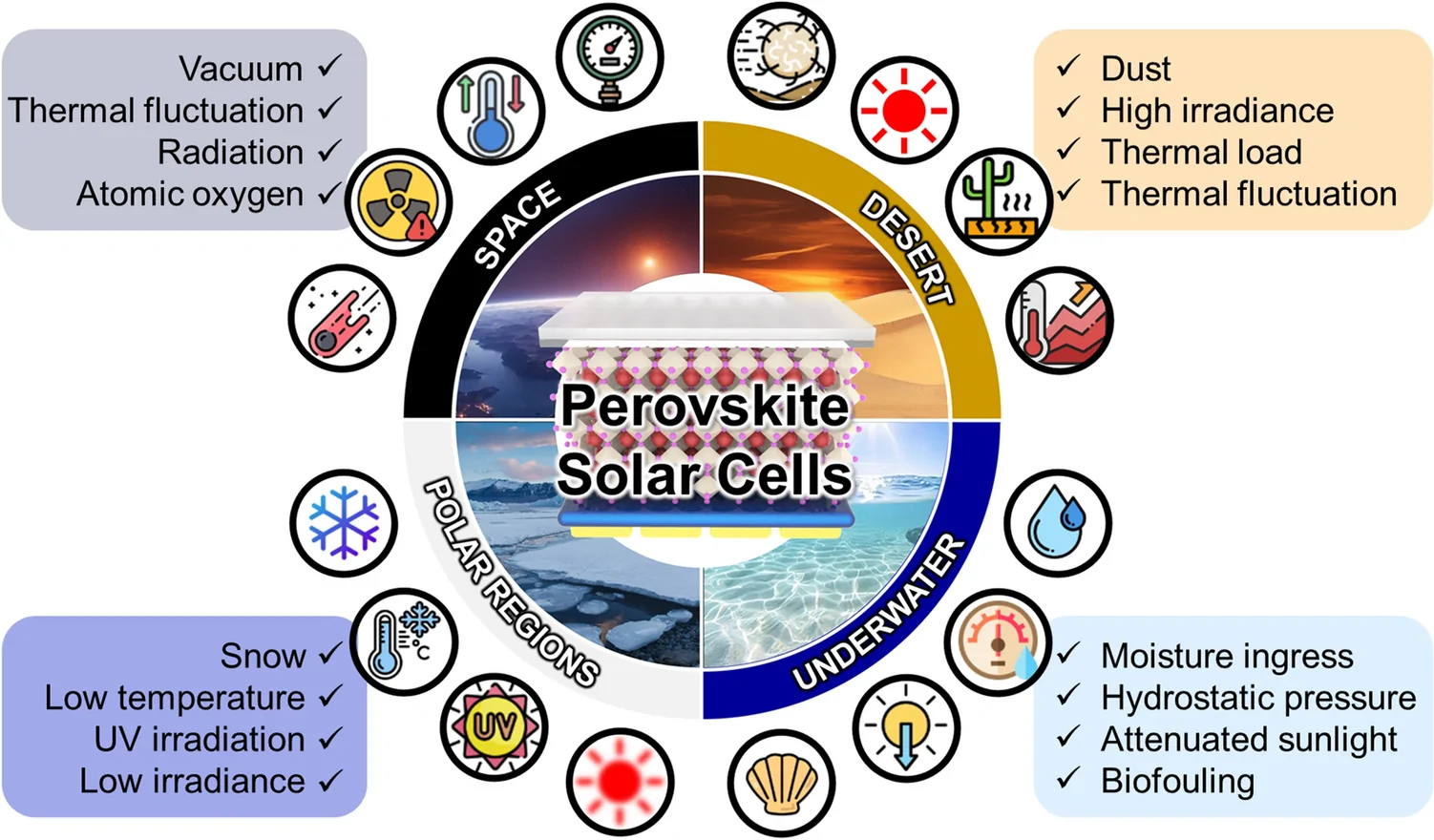
Schematic illustration of PSC operation under external stressors encountered in extreme environments

In this review, we examine the key environmental stressors that impact photovoltaic operation in extreme environments, including space, underwater, desert, polar, and high-altitude regions, as illustrated in Fig. 2. We further propose rational design principles for PSCs that encompass both material selection and device architecture. Our aim is to assess how PSCs can overcome the limitations of conventional photovoltaics and serve as reliable, self-sufficient power sources capable of supporting energy infrastructure under even the harshest environmental conditions.

## Understanding Extreme Environmental Conditions

To fully utilize PSCs in extreme environments both on Earth and beyond, it is essential to understand their behavior under diverse environmental conditions. This section aims to address the environmental variations and operational challenges faced in extreme settings, including outer space, underwater environments, deserts, polar regions, and high-altitude areas.

### Space Environment

Space lacks a power supply infrastructure, making independent energy sources indispensable for mission operations [23, 24]. Solar energy remains the most efficient and sustainable option, as it can be harvested in real time with virtually unlimited availability. Conventional space solar cells have predominantly relied on high-efficiency multijunction devices based on Si or group III–V semiconductors (e.g., GaAs, InGaP), owing to their excellent radiation resistance and long-standing record of reliable operation in orbit [6, 23, 25]. However, these technologies face limitations due to their high manufacturing costs, complex fabrication processes, significant mass and volume, and restricted substrate flexibility.

**Fig. 3:**
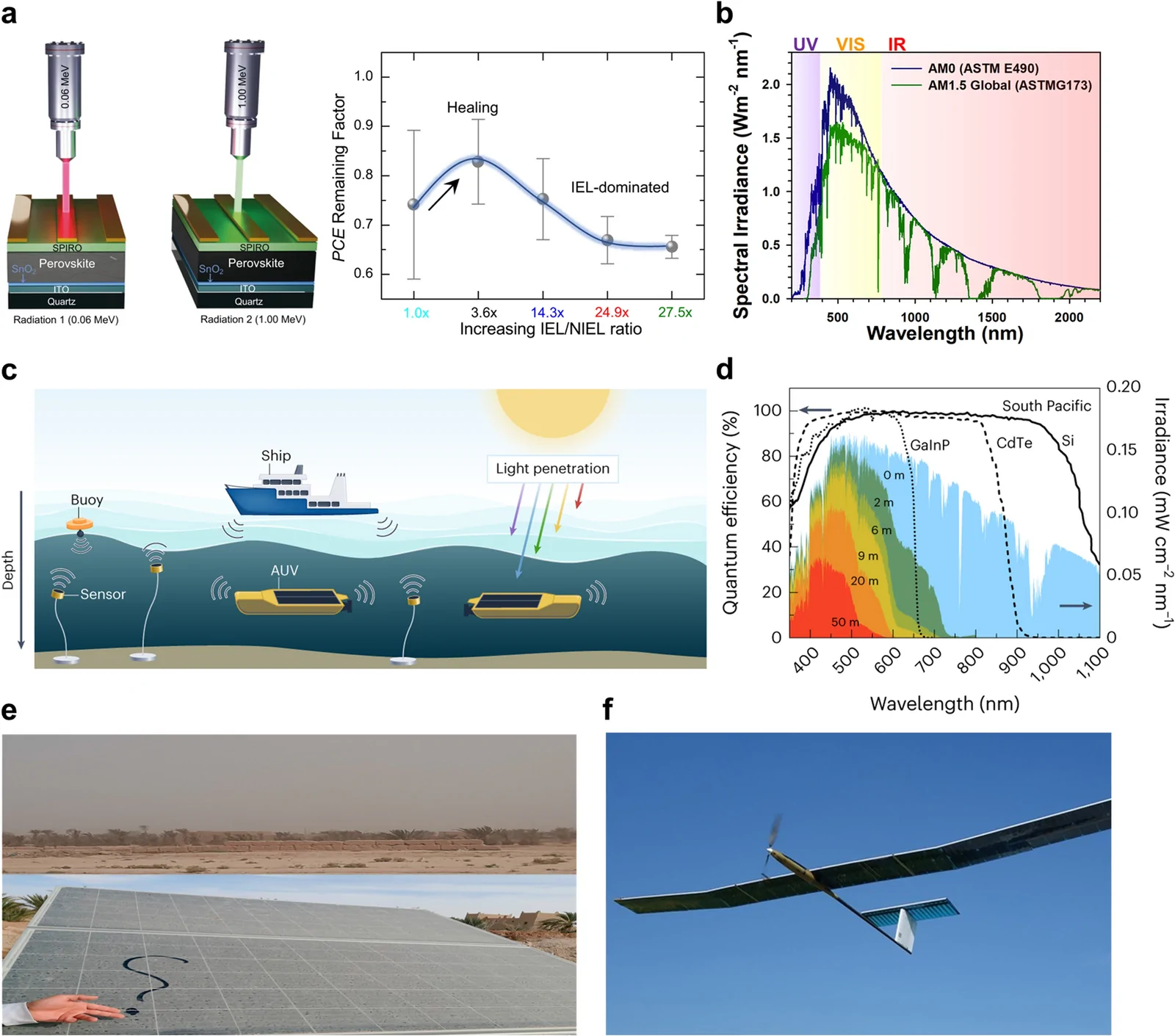
Environmental conditions relevant to extreme environments. **a** Schematic illustration of the dual-dose irradiation experiment used to investigate ionizing energy loss (IEL)-induced damage and recovery mechanisms in PSCs; corresponding PCE values are shown for PSCs under various IEL conditions [30]. Copyright 2025, Elsevier. **b** Schematic representation of spectral irradiance and wavelength distribution differences between AM0 and AM1.5G solar spectra [34]. **c** Schematic of an Internet of Underwater Things (IoUT) system. Solar cells engineered to absorb predominantly blue and green light are suited for high-efficiency underwater energy harvesting [59]. Copyright 2023, Springer Nature. **d** Simulated solar irradiance as a function of water depth based on the Beer–Lambert law applied to the AM1.5G spectrum, overlaid with the external quantum efficiency (EQE) curves for Si, CdTe, and GaInP solar cells [59]. Copyright 2023, Springer Nature. **e** Effect of wind-blown dust on photovoltaic panel performance [75]. Copyright 2015, John Wiley and Sons. **f** *AtlantikSolar* low-altitude, long-endurance (LALE) UAV is powered by solar energy [85]. Copyright 2017, John Wiley and Sons

In contrast, PSCs have recently emerged as promising next-generation solar cells for space applications, offering not only high performance but also advantages such as low weight and mechanical flexibility [11, 26–29]. Notably, recent studies have reported that perovskites may exhibit self-healing behavior under specific radiation conditions, attributed to their intrinsically soft lattice and strong polar nature, thereby enhancing their suitability as power sources in extraterrestrial environments [30–32]. For example, Kirmani et al. observed partial self-healing of perovskite layers under irradiation by high-energy protons, as illustrated in Fig. 3a [31]. Despite these promising findings, long-term operational data under the diverse and combined stressors present in actual space missions remain critically insufficient.

To be suitable for space applications, solar cells must meet several critical requirements [6]. High PCE is essential due to the limited available area for deployment. Devices must also exhibit strong resistance to radiation, UV irradiation, and atomic oxygen (AO), while maintaining excellent thermal stability and minimal weight. Currently, space-grade Si and III–V solar cells are evaluated using the AIAA S-111 standard [33], which requires tolerance to an electron fluence of 1 × 10^16^ cm^−2^ at 1 MeV and a proton fluence of 1 × 10^13^ cm^−2^ at 3 MeV. Additionally, devices must operate across a wide temperature range, from − 150 to 150 °C. While standardized evaluation protocols for PSCs have not yet been established, the development of such criteria will be essential for their integration into space power systems.

Solar radiation reaching the Earth’s surface is significantly attenuated due to atmospheric scattering and absorption, resulting in reduced incident energy compared to the space environment. Losses are particularly pronounced in the UV and infrared (IR) regions due to absorption by atmospheric constituents such as ozone, water vapor, and carbon dioxide [23, 24]. As a result, the total irradiance of the AM1.5G spectrum at the Earth’s surface is reduced to approximately 1000 W m^−2^, about 25%–30% lower than the extraterrestrial solar spectrum (Air Mass Zero, AM0), which has an irradiance of approximately 1367 W m^−2^.

In space, particularly in low Earth orbit (LEO) and geostationary orbit (GEO), the absence of an atmosphere allows solar radiation to reach devices without attenuation. Under these conditions, the solar spectrum is defined as AM0, which serves as the standard testing condition for space solar cells. As shown in Fig. 3b, the AM0 spectrum exhibits higher overall irradiance than the AM1.5G spectrum, with especially pronounced differences in the UV and IR regions [34, 35]. While terrestrial solar cells generally show low spectral response in these attenuated UV and IR regions, space solar cells must be designed to enhance light absorption across the full AM0 spectrum. Consequently, the development of PSCs for space applications requires careful consideration of AM0-specific conditions, including bandgap engineering, optical design, and stability under prolonged exposure to UV and IR irradiation.

In space, solar cells are subjected to significantly more extreme thermal cycling than on Earth. In LEO, they undergo rapid temperature fluctuations within a short orbital period of approximately 90 min, ranging from extreme cold (− 120 °C) to high temperatures (120 °C) [36]. This continuous thermal cycling imposes repeated thermal stress, causing frequent and severe expansion and contraction of materials—far beyond what is encountered under terrestrial conditions. Such thermal fluctuations induce mechanical stress due to mismatches in the coefficients of thermal expansion (CTE) among the device’s layers. These mismatches can lead to material defects, interfacial delamination, and eventual degradation in device performance [37, 38]. Therefore, to ensure reliable operation in the space environment, solar cells must be designed to withstand broader temperature ranges and more rapid thermal cycling than those typically experienced on Earth.

Radiation is one of the most defining and challenging aspects of the space environment compared with terrestrial conditions. Statistically, radiation-related effects account for nearly 40% of all malfunctions observed in space missions [39]. Understanding the nature and impact of this radiation is therefore essential, as it can cause substantial degradation of the semiconductor materials used in space solar cells [23]. The space radiation environment primarily consists of high-energy charged particles—mainly electrons and protons—emitted by the Sun and other celestial sources. These particles are trapped by Earth’s magnetic field, forming the Van Allen radiation belts, which extend from approximately 3000 to 40,000 km above the Earth’s surface [11, 40].

Most satellites in LEO operate within the inner Van Allen belt, where they are exposed to radiation composed mainly of electrons with energies ranging from 30 keV to 100 MeV and protons from 100 keV to 5 MeV [41]. When these energetic particles penetrate spacecraft materials, they undergo ionization and displacement processes, losing energy by transferring it to surrounding atoms. This energy transfer primarily generates Frenkel defects and simultaneously produces electron–hole pairs [42]. Such interactions can significantly degrade both the electrical and structural properties of semiconductor materials; when the accumulated fluence exceeds critical thresholds, device performance may be entirely compromised [43, 44]. Additionally, indirectly ionizing radiation, such as gamma rays and neutrons, can induce charge release and defect formation within materials [45, 46].

Atomic oxygen (AO), a highly reactive form of oxygen, is predominantly present in the LEO environment and is generated through the photodissociation of molecular oxygen (O_2_) by UV radiation [47]. In LEO, AO possesses a kinetic energy of approximately 5 eV and, upon interacting with material surfaces, can induce physical etching or chemical oxidation [48]. AO readily reacts with polymeric and carbon-based materials, typically producing CO and CO_2_ as reaction products. Since organic materials are commonly used as charge-transport or barrier layers in PSCs, their direct exposure to AO should be minimized or avoided altogether.

In addition, metallic and inorganic materials are susceptible to surface oxidation under AO exposure, which can lead to cracking or delamination, ultimately compromising device stability and performance over time [49]. To mitigate these effects, protective strategies such as thin inorganic coatings (e.g., Al_2_O_3_, SiO_*X*_) or self-healing coatings have been proposed and are considered essential for the reliable operation of solar cells in AO-rich environments [50].

The space environment is characterized by ultrahigh-vacuum (UHV) conditions; for instance, the atmospheric pressure at the altitude of the International Space Station (ISS) ranges from approximately 10^−5^ to 10^−6^ Pa [51]. Under such conditions, solar cells are subjected to complex mechanical, chemical, and thermal stresses far more severe than those encountered on Earth or in laboratory settings. In the absence of external pressure, residual solvents, moisture, and gaseous impurities trapped within materials can be rapidly released, leading to stress accumulation in films, interfacial delamination, and the formation of pinholes [52, 53]. These instabilities, particularly at the encapsulation layer or electrode–active layer interface, can significantly shorten device lifetime, necessitating the use of low-outgassing materials, vacuum preconditioning, and gas-barrier encapsulation films [54, 55].

Moreover, elevated temperatures in vacuum conditions can promote the volatilization of perovskite components (e.g., methylamine (MA), formamidine (FA), and hydrogen iodide (HI)), resulting in compositional inhomogeneity and defect formation [56, 57]. This degradation pathway may further exacerbate interactions with AO and solar wind particles in LEO. To mitigate such effects, the use of low-vapor-pressure perovskite compositions and volatilization-suppressing interfacial layers is recommended. Additionally, because convective heat transfer is negligible in a vacuum, localized overheating (hotspot formation) can occur in irradiated regions that are not efficiently cooled [58]. This issue is particularly critical for organic transport layers or glass substrates with low thermal conductivity; over time, such hotspots can accelerate both performance degradation and lifetime reduction. Accordingly, strategies such as employing high-thermal-conductivity substrates, radiative thermal management designs, and emissivity-controlled surfaces are essential for ensuring stable operation.

### Underwater Environment

Solar energy can be harnessed not only on the Earth’s surface but also underwater as a consistent power source. Although solar intensity decreases with depth, visible light—particularly within the blue-to-green spectral range (~ 450–550 nm)—penetrates relatively deeply into water [59, 60]. In the clearest ocean waters, sunlight can reach depths of up to ~ 50 m, where an irradiance exceeding 5 mW cm^−2^ (compared to 100 mW cm^−2^ at the Earth’s surface, AM1.5G) remains sufficient to power basic electronic devices [61]. These devices include Internet of Underwater Things (IoUT) systems, autonomous underwater vehicles (AUVs), and other submersible platforms (Fig. 3c) [59]. Moreover, underwater temperatures are generally stable within the range of 5 to 30 °C, which limits thermal stress and minimizes the impact of temperature fluctuations on device stability [59].

Nonetheless, the underwater environment inherently involves direct exposure to water, posing serious stability challenges for moisture- and ion-sensitive devices. In seawater, high salinity (Na^+^, Cl^−^, etc.), continuous moisture ingress, and organic contaminants from microbial activity can induce electrode corrosion, interfacial degradation, and decomposition of the photoactive layer—factors that critically undermine device lifetime and reliability. Therefore, the practical deployment of PSCs underwater requires more than incremental improvements in durability; it must incorporate comprehensive protection strategies such as chemical encapsulation, ion-blocking structures, and hydrophobic surface modification.

The underwater solar spectrum differs significantly from terrestrial AM1.5G conditions due to the wavelength-dependent attenuation of light by water. UV radiation is strongly scattered or absorbed within only a few centimeters, while IR radiation is rapidly absorbed by water molecules and dissipates within tens of centimeters, as shown in Fig. 3d. In contrast, visible light penetrates to much greater depths, with blue (~ 450 nm) and green (~ 525 nm) wavelengths traveling tens of meters. Consequently, the effective underwater spectrum is concentrated in the 450–550 nm range, whereas red light (> 650 nm) and UV radiation contribute minimally to power generation. Measurements indicate that in the clearest seawater, cyan wavelengths remain detectable at depths of approximately 50 m, with irradiance still exceeding 5 mW cm^−2^. This has direct implications for perovskite design: Compositions with a bandgap of 1.7–1.9 eV exhibit strong spectral overlap with the blue–green region, enabling efficient photovoltaic conversion under underwater conditions [62–64].

The primary stressor in the underwater environment is continuous and direct exposure to water. Perovskite materials are highly moisture-sensitive, and contact with water leads to rapid dissolution of the active layer, resulting in degradation of photovoltaic performance. Moreover, interfacial or charge-transport layers are particularly vulnerable, as moisture interferes with charge transport and introduces potential barriers. As a fundamental requirement, underwater PSCs demand pinhole-free encapsulation, impermeable barrier structures, and water-resistant interfacial engineering.

Seawater is rich in ionic species (Na^+^, Cl^−^, Mg^2+^, SO_4_^2−^, etc.), and their adsorption or penetration into the device can initiate electrochemical corrosion processes [65, 66]. Metallic electrodes (e.g., Ag, Cu) are particularly susceptible to potential shifts and ion diffusion, both of which compromise long-term stability [67]. In addition, salt ions may block charge-transport pathways or induce unintended doping effects. Organic components are also vulnerable to seawater exposure, where moisture uptake and salt-ion infiltration can trigger swelling, oxidative degradation, or variations in doping levels [68]. To mitigate these effects, ion-barrier encapsulation and hydrophobic surface coatings are essential.

With increasing depth, hydrostatic pressure rises by approximately 1 atm per 10 m. This pressure can deform encapsulation layers and substrates, leading to water ingress or interlayer delamination [69, 70]. Flexible polymer substrates are especially prone to such failure, as pressure fluctuations may cause stress accumulation and fatigue-induced cracking [71]. Consequently, structural design is critical for ensuring mechanical robustness under hydrostatic stress.

Long-term immersion in seawater exposes devices to microbial activity, algae, and organic matter, resulting in biofouling [72, 73]. This phenomenon reduces light transmission and leads to the formation of electrochemically active films on device surfaces, thereby degrading both optical and electrical performance. Certain microorganisms further exacerbate degradation by secreting acidic metabolites or oxidative enzymes that attack encapsulants and accelerate interfacial corrosion. To mitigate these effects, strategies such as antifouling coatings, self-cleaning surface architectures, and photocatalytic biobarriers have been proposed.

### Terrestrial Extreme Environments

Even on Earth’s surface, certain regions constitute extreme environments, characterized by high or low temperatures, intense UV radiation, strong winds, low atmospheric pressure, and large diurnal temperature fluctuations. These conditions often exceed the operational limits of conventional terrestrial photovoltaic systems. Although the high efficiency of PSCs indicates strong potential for deployment in such regions, achieving sufficient environmental resilience against diverse climatic and mechanical stressors remains essential.

#### Deserts

Deserts represent one of the most demanding terrestrial environments, where solar cells are exposed to elevated temperatures (> 40 °C), high solar irradiance (> 1000 W m^−2^), low relative humidity (< 10%), sandstorms, dust accumulation, and sharp diurnal temperature fluctuations [74, 75]. Ground temperatures in the Sahara Desert can reach up to 70 °C, with ambient air temperatures rising to approximately 50 °C [76, 77]. Intense UV exposure and thermal stress can degrade the photothermal stability of perovskites, often resulting in decomposition or phase transitions. Additionally, large temperature swings induce interfacial stress due to mismatch in the CTE, leading to delamination or microcrack formation. Dust and sand particles further contribute to performance degradation through optical shading, surface erosion, and increased reflection losses, thereby reducing the photon-to-current yield (Fig. 3e) [75]. To ensure reliable PSC operation in desert conditions, approaches such as UV-blocking films, thermally stable material compositions, hydrophobic and antisoiling coatings, and mechanically flexible device architectures are required.

#### Polar Regions

The Arctic and Antarctic regions are characterized by extreme cold, with temperatures frequently below − 30 °C, in addition to snow, ice, frost, and diffuse sunlight due to low solar elevation angles [78]. Such ultralow temperatures hinder carrier transport and extraction, increasing series resistance and reducing the fill factor (FF) of devices [79]. Moreover, thermal expansion mismatch between the perovskite and adjacent layers can lead to interfacial defects and long-term degradation [80]. Interestingly, perovskites demonstrate certain optoelectronic advantages at low temperatures [81, 82]. For instance, the cubic-to-tetragonal phase transition can facilitate the self-elimination of intrinsic defect states through thermodynamically and kinetically driven processes. From this perspective, PSCs offer potential for efficient light harvesting under cryogenic conditions, provided that interfacial compatibility and charge transport are carefully engineered. Additionally, snow and ice accumulation on module surfaces poses a practical challenge in polar environments, as it can severely reduce optical transmission and intermittently block incident light [83, 84]. In this context, vertical or near-vertical panel orientations, which are commonly adopted in polar PV installations, can mitigate snow accumulation while simultaneously improving light capture under low solar elevation angles. Such orientation-dependent considerations, together with surface coatings that suppress ice adhesion or promote self-cleaning, may play an important role in enabling reliable PSC operation in polar regions. Nevertheless, practical deployment in polar environments remains limited by repeated freeze–thaw cycles, thermomechanical stress, low solar irradiance, and surface icing, all of which demand further technological solutions.

#### High-Altitude Regions

At high altitudes, solar power is employed for unmanned aerial vehicles (UAVs), meteorological balloons, and off-grid energy supply for high-mountain facilities (Fig. 3f) [85]. Above 3000 m, the environment is characterized by low atmospheric pressure (~ 0.7 atm or lower), intense UV radiation (UV-A and UV-B), sharp fluctuations in solar irradiance, low temperatures, and dry air [86, 87]. Reduced pressure enhances gas permeation and degassing within encapsulation layers, promoting delamination due to mechanical stress [88]. Moreover, UV intensity increases by approximately 10%–20% compared with sea level, accelerating photooxidative degradation of the perovskite absorber, charge-transport layers, and encapsulants [89, 90]. In combination with strong irradiance and temperature variation, these factors impose repeated interfacial thermomechanical stress, photothermal expansion, and optical reflection losses. To ensure stable PSC operation in high-altitude regions, critical strategies include low-pressure-tolerant encapsulation, enhanced UV stability, and stress-relief device architectures.

### Key Considerations Across Extreme Environments

**Fig. 4:**
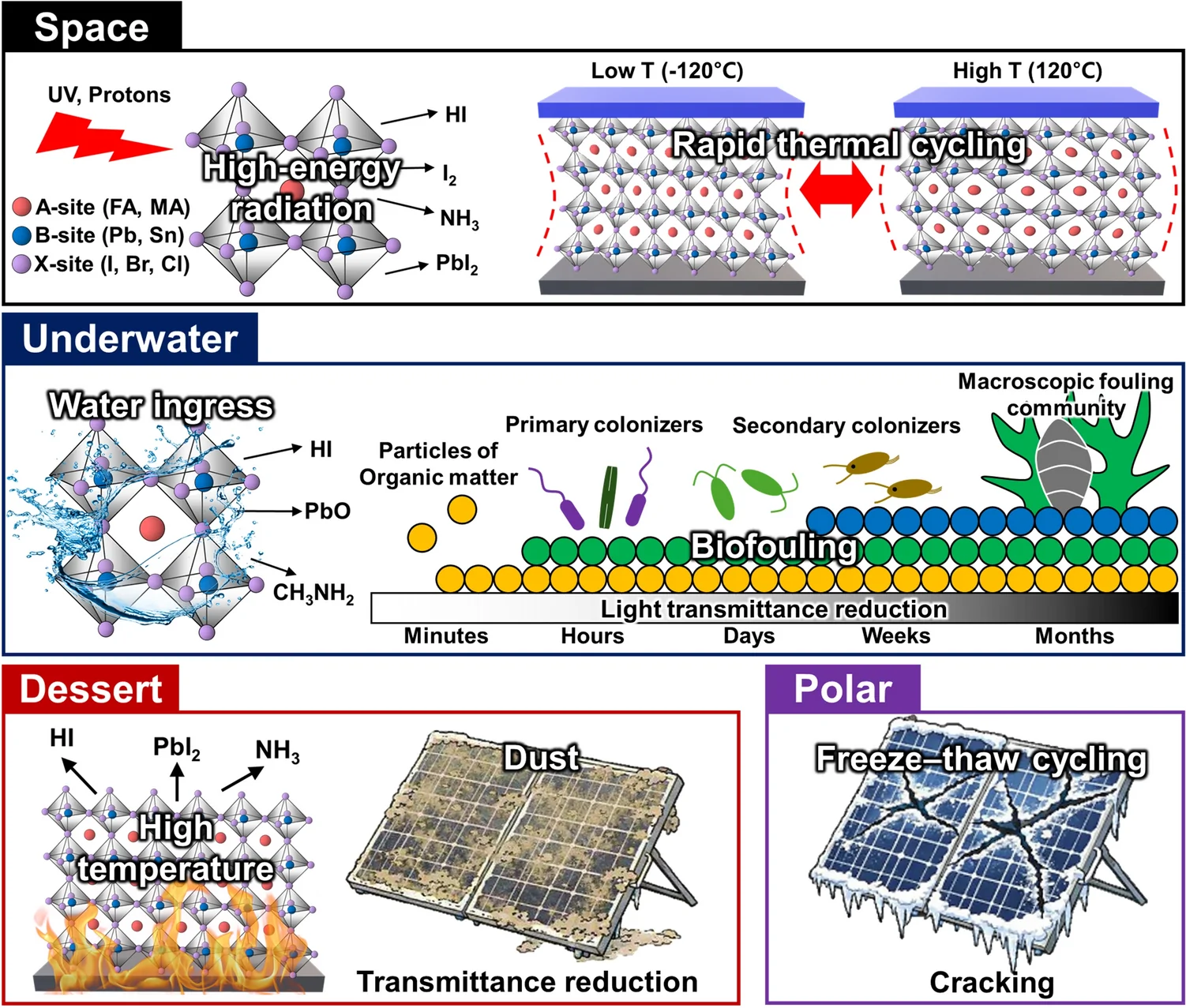
Schematic illustration of key degradation mechanisms of PSCs across various extreme environmental conditions

PSCs are subjected to a diverse range of stressors depending on the deployment environment, including extreme thermal cycling and radiation in space; saline exposure and biofouling in underwater settings; intense UV radiation and dust in desert regions; cryogenic temperatures and freeze–thaw cycles in polar climates; and low pressure combined with high UV intensity at high altitudes. To underscore the unique operating conditions and dominant degradation pathways in each scenario, Table 1 and Fig. 4 summarize the key environmental factors that must be considered when designing PSCs for practical applications in extreme environments.

**Table 1:** Representative environmental stressors characteristic of various extreme environments

Environment	Temperature conditions	Pressure conditions	Radiation/light stress	Chemical/mechanical stress
Space (LEO, etc.)	− 120 °C to 120 °C (thermal cycling)	Ultrahigh vacuum (~ 10^−6^ Torr)	High-energy particles (protons, electrons, gamma), UV	Atomic oxygen (AO), plasma impact, micrometeoroids, mechanical shock/vibration
Underwater (surface–shallow depth)	+ 5 °C to + 30 °C (seasonal/latitudinal variations)	Atmospheric to mild hydrostatic (1–5 atm)	Attenuated sunlight and UV (depth-dependent)	Salinity, humidity, waves, and mechanical impact, biofouling
Desert	10 °C to 40 °C (large diurnal swings)	Atmospheric pressure	Intense sunlight and UV	Sand/dust, abrasion, surface erosion, thermomechanical stress
Polar regions	− 60 °C to 0 °C	Atmospheric pressure	Scattered/reflected UV, low irradiance	Frost, snow, ice; repeated freeze–thaw cycles; thermal stress
High-altitude	− 20 °C to + 25 °C	Low pressure (~ 0.7 atm)	Strong irradiance and UV	Frost, encapsulant shrinkage, and degassing under reduced pressure

## Overcoming Stress Factors in PSCs

This chapter examines the major stress factors—light, temperature, and water—that affect PSCs in both terrestrial and extraterrestrial extreme environments, and provides an in-depth discussion of strategies to mitigate their impact. While most of the stabilization strategies discussed in this chapter were developed and evaluated under laboratory-based or accelerated test conditions, they address the same fundamental degradation mechanisms that govern device failure in real-world extreme environments. Thus, these approaches provide generalizable design principles for improving the stability of PSCs, rather than being limited to specific testing scenarios.

### Light Stress

#### Degradation Pathways of PSCs under Light Stress

In the early stages of PSC research, various strategies were explored to address photoinstability, including compositional engineering, device architecture optimization, and interfacial engineering. These approaches have led to notable advancements, with many recent studies reporting operational stability exceeding 1000 h under continuous illumination with maximum-power-point tracking (MPPT) conditions [16–18]. Consequently, PSCs based on current technology can be considered stable under general light exposure. However, it is important to note that most stability assessments have utilized LED-based light sources, which lack sufficient UV content. As a result, while PSCs are now regarded as reasonably stable under visible-light illumination, their durability under high-energy UV irradiation remains inadequately characterized. Recent studies have demonstrated that exposure to UV-rich outdoor sunlight induces more severe degradation than conventional LED-based testing methods [91–93]. This discrepancy underscores a critical limitation, as environments with high UV intensity—such as outer space or high-altitude regions—demand more rigorous evaluation of PSC stability under such conditions.

Several studies have reported the degradation pathways of perovskites under UV irradiation. When photons with energies exceeding the bandgap of PbI_2_ (2.34 eV, ~ 530 nm) are absorbed, PbI_2_ can decompose into metallic Pb^0^ and volatile I_2_, as represented in Eq. (1) [89, 94].

1 $$ {\mathrm{CH}}_{3} {\mathrm{NH}}_{3} {\mathrm{PbI}}_{3} \mathop \leftrightarrow \limits^{{{{hv}}}} {\mathrm{PbI}}_{2} \left( {\mathrm{s}} \right) + {\mathrm{Pb}} \left( {\mathrm{s}} \right) + {\mathrm{I}}_{2} { }\left( {\mathrm{g}} \right) + {\mathrm{CH}}_{3} {\mathrm{NH}}_{2} \left( {\mathrm{g}} \right) + {\mathrm{CH}}_{3} {\mathrm{I}} \left( {\mathrm{g}} \right) + {\mathrm{HI}} \left( {\mathrm{g}} \right) + {\mathrm{NH}}_{3} \left( {\mathrm{g}} \right) $$

The presence of PbI_2_ in perovskite films is often attributed to incomplete conversion during fabrication or the loss of A-site cations under external stress. The I_2_ produced can trigger further degradation of iodide-based perovskites through a self-amplifying chemical chain reaction [95], leading to accelerated decomposition under prolonged light exposure. This degradation is further exacerbated in PSCs that incorporate metal oxide transport layers with photocatalytic activity, such as TiO_2_, where UV irradiation promotes iodide oxidation and Pb^2+^ reduction [94]. Additionally, illumination of MAPbI_3_ with photon energies above ~ 450 nm has been shown to release CH_3_NH_2_ and H_2_, suggesting cleavage of N–H bonds under sunlight [96]. Collectively, these findings demonstrate that PSCs are intrinsically susceptible to degradation under high-energy UV exposure. Therefore, the development of effective mitigation strategies is essential for their reliable operation in extreme environments.

**Fig. 5:**
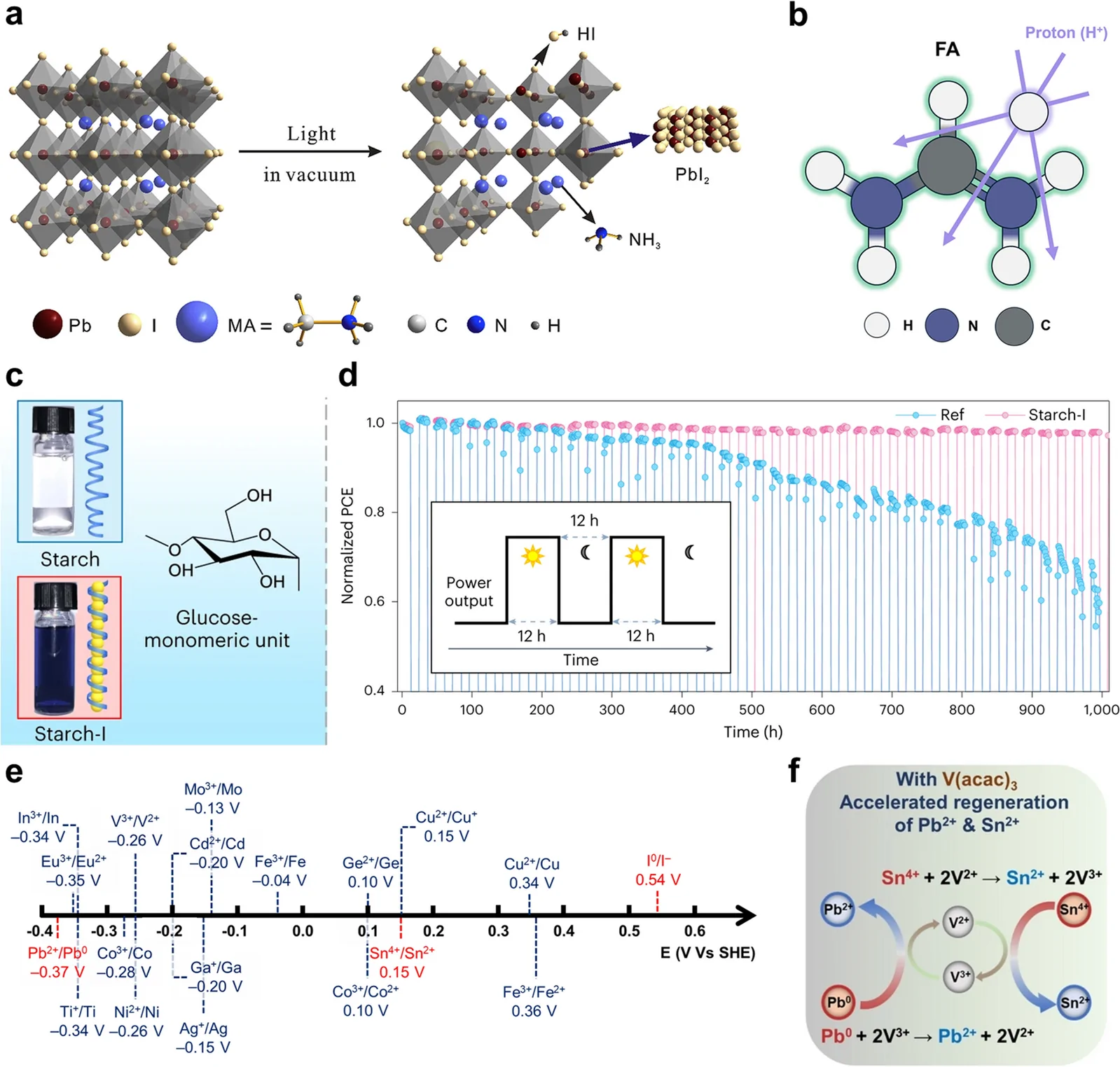
Irradiation-induced degradation pathways in PSCs and corresponding mitigation strategies.** a** Schematic illustration of light-induced degradation mechanisms in a CH_3_NH_3_PbI_3_ perovskite film [97]. Copyright 2019, Elsevier. **b** Schematic showing the FA molecule and potential bond cleavage triggered by proton irradiation [99]. Copyright 2025, Elsevier. **c** Photographs of starch and Starch-I solutions alongside the molecular structure of Starch-I [119]. **d** Simulated tracking performance of reference and Starch-I devices over a 24 h diurnal cycle [119]. Copyright 2023, Springer Nature. **e** Standard redox potentials of selected ionic pairs. **f** Proposed mechanism illustrating how the V^3+^/V^2+^ redox shuttle accelerates self-healing redox reactions in Sn–Pb perovskites [123]. Copyright 2023, John Wiley & Sons

It should also be noted that, despite the reasonable stability of perovskites under white-light exposure, irreversible decomposition can occur under vacuum conditions. Hong et al. reported that MAPbI_3_ perovskite films exposed to LED illumination under high-vacuum conditions (< 1 × 10^−8^ Torr) underwent irreversible degradation [97]. Initially, MAPbI_3_ decomposes into MAI and PbI_2_, followed by further decomposition of PbI_2_ into metallic Pb and I_2_ (Fig. 5a). This degradation process saturates after approximately 24 h of illumination, exhibiting a self-limiting behavior. The resulting decomposition products can remain physically adsorbed on the perovskite surface, forming a barrier layer that suppresses further degradation of the underlying material. However, this insulating layer induces interfacial band bending, hinders carrier transport, and ultimately deteriorates device performance. The corresponding degradation reactions are described by Eqs. (2) and (3):

2 $$  {\mathrm{CH}}_{3} {\mathrm{NH}}_{3} {\mathrm{PbI}}_{3}  + hv\,\xrightarrow{{{\mathrm{UHV}}}}\,~{\mathrm{CH}}_{3} {\mathrm{I}}~\left( {\mathrm{g}} \right) + {\mathrm{NH}}_{3} ~\left( {\mathrm{g}} \right) + ~{\mathrm{PbI}}_{2} ~\left( s \right) + {\mathrm{HI}}~\left( {\mathrm{g}} \right) + {\mathrm{CH}}_{3} {\mathrm{NH}}_{2} ~\left( {\mathrm{g}} \right)  $$

3 $$  {\mathrm{PbI}}_{2}  + hv\,\xrightarrow{{{\mathrm{UHV}}}}\,{\mathrm{Pb}}~\left( {\mathrm{s}} \right) + {\mathrm{I}}_{2} ~\left( {\mathrm{s}} \right)  $$

In space environments, the effects of ionizing radiation, including protons, X-rays, and γ-rays, on PSCs have also been investigated. Notably, perovskites exhibit distinct interaction mechanisms under irradiation compared to conventional semiconductors. Experimental studies have shown that PSCs can withstand proton irradiation doses approximately 100 times higher than those tolerated by Si or III–V compound semiconductors [45, 98]. Although the precise mechanisms remain under investigation, one plausible explanation lies in the intrinsically thin perovskite absorber layer, which reduces the effective volume available for radiation interactions [45, 98]. Because radiation-induced damage is related to the interaction path length, a thinner layer results in a shorter traversal distance and less accumulated damage. Additionally, perovskites display self-healing behavior under irradiation, potentially due to a combination of their soft lattice structure, strong electron–phonon coupling, and low intrinsic thermal conductivity [30].

Nevertheless, complete recovery of device performance is not achieved, primarily due to the irreversible decomposition of organic components. Shim et al*.* reported the degradation of FA cations in FAPbI_3_ under proton irradiation [99]. Specifically, incident protons cleave C–H and N–H bonds within the FA molecule, generating H^+^ radicals (Fig. 5b). Notably, N–H bond cleavage leads to the deprotonation of the FA cation, forming hydrogen halides (HX, where X = Cl, Br, I) and FA. These intermediates undergo irreversible reactions, producing *sym*-triazine (C_3_H_3_N_3_) and ammonia, which ultimately contribute to device degradation, as described in Eqs. (4–7):

4 $$ {\mathrm{CH}}({\mathrm{NH}}_{2} )_{2}^{ + } + {\mathrm{H}}^{ + } \, \to \,{\text{ CH}}\left( {{\mathrm{NH}}} \right){\mathrm{NH}}_{2} + {\mathrm{H}}^{ + } $$

5 $$ 3\left( {{\mathrm{CH}}\left( {{\mathrm{NH}}} \right){\mathrm{NH}}_{2} } \right)\, \to \,{\mathrm{C}}_{3} {\mathrm{H}}_{3} {\mathrm{N}}_{3} + 3{\mathrm{NH}}_{3} \left( {\mathrm{g}} \right) $$

6 $$ {\mathrm{H}}^{ + } + {\mathrm{X}}\, \to \, {\mathrm{HX}} \left( {\mathrm{g}} \right) $$

7 $$ 2{\mathrm{H}}^{ + } \, \to \,{\mathrm{H}}_{2} \left( {\mathrm{g}} \right) $$

Additionally, C–N bond cleavage may lead to the formation of ammonia and MA cations (CH_3_NH_2_^+^). These species are reported to exhibit high volatility and mobility due to their interactions with H^+^ radicals, as further detailed in Eqs. (8–10):

8 $$ {\mathrm{CH}}({\mathrm{NH}}_{2} )_{2}^{ + } + {\mathrm{H}}^{ + } \, \to \,{\mathrm{CHNH}}_{2}^{ \bullet } + {\mathrm{NH}}_{2}^{ \bullet } $$

9 $$ {\mathrm{CHNH}}_{2}^{ \bullet } + 2{\mathrm{H}}^{ + } \, \to \,{\mathrm{CH}}_{3} {\mathrm{NH}}_{2} \left( {\mathrm{g}} \right) $$

10 $$ {\mathrm{NH}}_{2}^{ \bullet } + {\mathrm{H}}^{ + } \, \to \,{\mathrm{NH}}_{3} \left( {\mathrm{g}} \right){ } $$

Through these reaction pathways, FA can be irreversibly decomposed into products such as *sym*-triazine and volatile species (NH_3_, HI, H_2_), thereby depleting the A-site cation content relative to the inorganic constituents (Pb, I). This imbalance promotes the formation of non-photoactive phases such as the δ-phase and PbI_2_.

#### Approaches for Mitigating Photodegradation

Improving photostability requires fabricating high-quality perovskite films with minimal defects, which otherwise serve as pathways for halide and cation migration during device operation [100–103]. These defects are primarily located at grain boundaries and surfaces, where passivation can effectively suppress ion migration [104]. Accordingly, various approaches have been developed to passivate these defect sites. Surface passivation commonly employs alkylammonium chains, cyclic or aromatic ammonium cations, and ammonium ligand salts containing halide anions [19]. The ammonium cations in these salts can interact with the perovskite surface through A-site vacancies or hydrogen bonding, forming a thin molecular layer atop the perovskite film.

Under certain conditions, bulky ammonium cations may partially substitute A-site cations, leading to surface reconstruction and the formation of lower-dimensional perovskite structures [105]. Such surface reconstruction typically results in 2D perovskite capping layers on 3D perovskite absorbers, forming 2D/3D heterostructures. In these heterostructures, the surface-localized 2D phase passivates surface and grain-boundary defects while restricting ion migration across the interface. These effects are accompanied by suppressed non-radiative recombination under continuous illumination [106, 107]. As a result, 2D/3D PSCs can exhibit improved operational stability under prolonged, high-flux illumination and environmental stress by limiting degradation pathways relevant to extreme operating conditions.

For grain-boundary passivation, a common strategy involves incorporating passivating agents directly into the perovskite precursor solution. These additives include ammonium salts similar to those used in surface treatments, as well as excess PbI_2_, metal cations, anions, and polymers [108]. Another approach to reduce grain-boundary defects is to increase grain size, thereby decreasing boundary density. This can be achieved through substrate surface modification, additive engineering, and the controlled regulation of solvent evaporation and crystallization kinetics [109–111].

Additionally, recent studies have shown that strengthening the chemical bonding at the bottom perovskite interface is an effective strategy for suppressing light-induced degradation [16, 91, 112]. By introducing interfacial molecules capable of multidentate coordination or strong anchoring interactions, the bonding strength between the perovskite absorber and the underlying electrode or charge-transport layer can be significantly enhanced. Such reinforced interfaces reduce the density of interfacial trap states that act as initiation sites for photoinduced ion migration under illumination. Moreover, strong interfacial bonding suppresses the migration of photoactivated halide ions and A-site cations toward the bottom interface, thereby mitigating irreversible interfacial decomposition and non-radiative recombination losses during prolonged light exposure.

In particular, Fei et al. introduced an aromatic phosphonic acid-based molecule, [2-(9-ethyl-9H-carbazol-3-yl)ethyl]phosphonic acid (EtCz3EPA), at the perovskite/ITO interface to form strong chemical bonding [91]. Devices incorporating this interfacial modifier exhibited a *T*_90_ lifetime of 1780 h under illumination containing ~ 3.5% UV, whereas the reference devices showed a *T*_90_ lifetime of only 190 h. These results clearly demonstrate that controlling bonding strength at the bottom interface is a critical design principle for achieving photostability in UV-containing and extreme-illumination environments.

In addition to interfacial bonding control, photostability can be directly improved by managing UV photons through charge-transport layers (CTLs) engineering and UV filtering. In early PSC research, TiO_2_ was predominantly employed as the ETL. However, TiO_2_ exhibits photocatalytic activity under UV irradiation, accelerating the photodegradation of perovskites [113]. To mitigate this issue, alternative ETLs that are less photocatalytically active under UV illumination, such as SnO_2_ and oxide perovskites, have been introduced. For example, Shin et al. reported that PSCs employing a lanthanum (La)-doped BaSnO_3_ ETL retained 93.3% of their initial PCE after 1000 h of UV-containing illumination, whereas TiO_2_-based devices suffered complete degradation within 500 h under identical conditions [114].

In parallel, UV filtering has emerged as a general and straightforward strategy to suppress UV-induced photocatalytic activity in ETLs. Yoon et al. introduced a TiO_2_ nanoparticle/graphene nanodot (TiO_2_ NPs/GNDs) composite layer on the front side of the FTO glass to selectively absorb UV photons [115]. As a result, PSCs incorporating the TiO_2_ NPs/GNDs exhibited only a 15% PCE loss after 100 h of UV irradiation, whereas reference devices with a TiO_2_ absorber layer and those without any UV-absorbing layer showed 70% and 92% degradation, respectively.

To further enhance the photostability of PSCs, one promising strategy is to exploit their intrinsic self-healing behavior under light-induced stress. Bag et al. reported the quasi-reversibility of photoinduced degradation phenomena driven by ion migration, observing that MAPbI_3_-based devices recovered most of their PCE after approximately 15 min in the dark [116]. Subsequently, Nie et al. proposed that this self-healing behavior originates from the formation of light-activated metastable deep-level trap states [117]. Under illumination, these metastable states accumulate over tens of minutes to hours, leading to the development of charged regions within the perovskite film and causing a reduction in photocurrent. However, when the device is kept in the dark, most of these light-activated trap states dissipate, allowing the photocurrent to nearly fully recover upon reillumination. Cappel et al. further suggested that the conversion of PbI_2_ into metallic Pb^0^ is reversible [118]. Through this reaction, Pb^0^ can recombine with surrounding I_2_ and FAI to regenerate the perovskite structure. These findings indicate that self-healing is feasible if the loss of volatile species is prevented. This implies that, in UHV environments, such as outer space, developing technologies to retain volatile species may be an effective strategy for improving photostability.

Zhang et al. investigated the reversibility of I^−^ migration, a primary contributor to performance degradation during device operation [119]. Building on this understanding, they proposed a strategy to more effectively harness the material’s self-healing behavior by incorporating a starch–polyiodide (Starch-I) supermolecule at the buried interface of the device (Glass/ITO/SnO_2_/Starch-I/Perovskite/Spiro-OMeTAD/Au). Device stability was evaluated under a 24 h diurnal cycle (12 h light/12 h dark) (Fig. 5c). As shown in Fig. 5d, PSCs containing Starch-I retained more than 98% of their initial PCE after 42 aging cycles, whereas control devices without Starch-I exhibited approximately 40% PCE loss. This improvement was attributed to the Starch-I layer’s ability to release additional I^−^ ions, which neutralize iodide vacancy defects in the perovskite while concurrently suppressing further I^−^ migration. Notably, stability evaluation under diurnal light–dark cycling is particularly important, as such conditions better reflect realistic operating environments and allow for the recovery of ion migration-induced degradation during dark periods [120, 121].

Another self-healing approach involves the use of redox couples that can reversibly oxidize or reduce degradation products within the perovskite, thereby restoring their original chemical states [122]. When the redox potential of the couple lies between the standard redox potentials (E^0^) of the relevant degradation species, both oxidation and reduction reactions become thermodynamically favorable, enhancing the overall reaction kinetics. Figure 5e illustrates the redox potentials of typical perovskite degradation products (Pb^0^, Sn^4+^, I_2_) alongside various metal-based redox couples [122, 123]. Several transition metals and lanthanides possess redox potentials well suited to mediating electron transfer between Pb^0^ and I_2_. Redox couples with potentials located between Pb^0^/Pb^2+^ and I_2_/2I^−^ can facilitate reversible electron exchange, effectively acting as redox shuttles that promote the reformation of PbI_2_.

Wang et al. first introduced the concept of a Eu^2+^/Eu^3+^ (E^0^ = − 0.35 V) redox shuttle in PSCs [124]. Within the perovskite film, Eu ions function as catalytic self-healing agents during operation by oxidizing Pb^0^ and reducing I_2_. As a result, the device retained 93% of its initial PCE after 100 h of continuous 1-sun illumination. Additionally, the Eu^2+^/Eu^3+^ redox shuttle mitigated radiation-induced degradation [125]. Other researchers have investigated a variety of materials as potential redox shuttles in perovskite systems, including GdF_3_, ferrocene, I^−^/I_3_^−^, and polyoxometalates [126–130]. This redox-based self-healing strategy has also been extended to Sn-based perovskites, where Sn^2+^ is inherently susceptible to oxidation into Sn^4+^ due to its thermodynamic instability. He et al. demonstrated that the reaction between photoreduced Pb^0^ species and Sn^4+^ is thermodynamically favorable (Eq. (11)) [123]:

11 $$ {\mathrm{Pb}}^{0} + {\mathrm{Sn}}^{4 + } \, \to \,{\mathrm{Pb}}^{2 + } + {\mathrm{Sn}}^{2 + } \quad \Delta G < 0 $$

The spontaneity of this process can be rationalized using redox potentials: Species with lower standard reduction potentials are more readily oxidized. Considering the standard potentials for Sn^4+^/Sn^2+^ (+ 0.151 V) and Pb^2+^/Pb^0^ (− 0.365 V), Pb^0^ is relatively prone to oxidation, while Sn^4+^ is more easily reduced, supporting the thermodynamic feasibility of this redox reaction. To further accelerate the process, a V^3+^/V^2+^ redox couple (E^0^ = − 0.26 V) was introduced, which facilitated the regeneration of both Sn^2+^ and Pb^2+^ in Sn/Pb mixed perovskites, thereby significantly enhancing photostability (Fig. 5f).

These self-healing strategies may also be applicable in extreme environments, where perovskite devices are exposed to intense radiation and vacuum conditions. In such settings, preventing the loss of volatile species generated during photodegradation is critical to maintaining the self-healing process. Therefore, the development of advanced physical and chemical encapsulation technologies capable of effectively suppressing volatile loss is essential. When combined with photostability-enhancing strategies, such encapsulation approaches may provide a synergistic route toward improving the long-term operational stability of PSCs under harsh environmental conditions. Representative light management strategies for enhancing PSC stability are summarized in Table 2.

**Table 2:** Representative light management strategies for improving stability of PSCs

Light management strategies	PCE (%)	Measurement conditions	Stability	References
Surface passivation with dimethylphenethylsulfonium iodide	23.3	LED, N_2_	> 99% PCE retention after 4500 h	[18]
2D (BA_2_FAPb_2_I_7_)-stabilized FAPbI_3_ devices	24.1	AM1.5G, 85°C	> 97% PCE retention after 1000 h	[17]
Hybrid SAM (4,4′,4″-nitrilotribenzoic acid)	26.2	white-light LED, 65°C	96.1% PCE retention after 2400 h	[16]
Hybrid SAM ([2-(9-ethyl-9H-carbazol-3-yl)ethyl]phosphonic acid)	23.1	Light emission plasma (LEP) lamp (3.5% UV light), 60°C	> 90% PCE retention after 1780 h	[91]
UV-inactive ETL (La:BaSnO_3_)	21.2	AM 1.5G	> 93% PCE retention after 1000 h	[114]
UV filter (TiO_2_ NPs/graphene)	16.7	UV irradiation (365 nm)	> 85% PCE retention after 100 h	[115]
Vacuum operation	-	LED, < 1 × 10^−8^ Torr	Irreversible degradation; degradation saturates ~ 24 h	[97]
Self-healing by interface ion reservoir (Starch-I)	24.3	24 h diurnal cycle (12 h light / 12 h dark)	> 90% PCE retention after 42 cycles	[119]
Redox-shuttle (Eu^2+^/Eu^3+^)	21.5	AM1.5G	> 93% PCE retention after 1000 h	[124]
Redox-shuttle (V^2+^/V^3+^)	21.2	AM1.5G, N_2_	> 90% PCE retention after 1000 h	[123]

### Temperature

#### Degradation Pathways of PSCs Under Thermal Stress

An important pathway for thermal degradation in PSCs involves the generation of volatile species from the perovskite, which becomes observable at relatively low temperatures (as low as 85 °C). These volatile compounds are released from the perovskite films under thermal stress and ambient factors such as oxygen and humidity, leaving predominantly inorganic residues (e.g., PbI_2_) that are inadequate for photovoltaic conversion. This degradation behavior is attributed to the soft nature of perovskites, which exhibit a low formation enthalpy—approximately 0.1 eV per formula unit for MAPbI_3_. The thermal decomposition reactions of MAPbI_3_ are summarized as follows (Eqs. (12) and (13)) [131]:

12 $$ {\mathrm{CH}}_{3} {\mathrm{NH}}_{3} {\mathrm{PbI}}_{3} \left( {\mathrm{s}} \right)\, \leftrightarrow \,{\mathrm{CH}}_{3} {\mathrm{NH}}_{2} \left( {\mathrm{g}} \right) + {\mathrm{HI}} \left( {\mathrm{g}} \right) + {\mathrm{PbI}}_{2} \left( {\mathrm{s}} \right) $$

13 $$ {\mathrm{CH}}_{3} {\mathrm{NH}}_{3} {\mathrm{PbI}}_{3} \left( {\mathrm{s}} \right)\, \to \,{\mathrm{NH}}_{3} \left( {\mathrm{g}} \right) + {\mathrm{CH}}_{3} {\mathrm{I}} \left( {\mathrm{g}} \right) + {\mathrm{PbI}}_{2} \left( {\mathrm{s}} \right) $$

Although the dominant degradation pathway depends on the surrounding environment, the formation of CH_3_I and NH_3_ via Eq. (13) requires cleavage of the strong C–N bond, which entails a high activation energy. Consequently, at relatively low temperatures (55–85 °C), decomposition is more likely to proceed via the reversible pathway described in Eq. (12) [132].

**Fig. 6:**
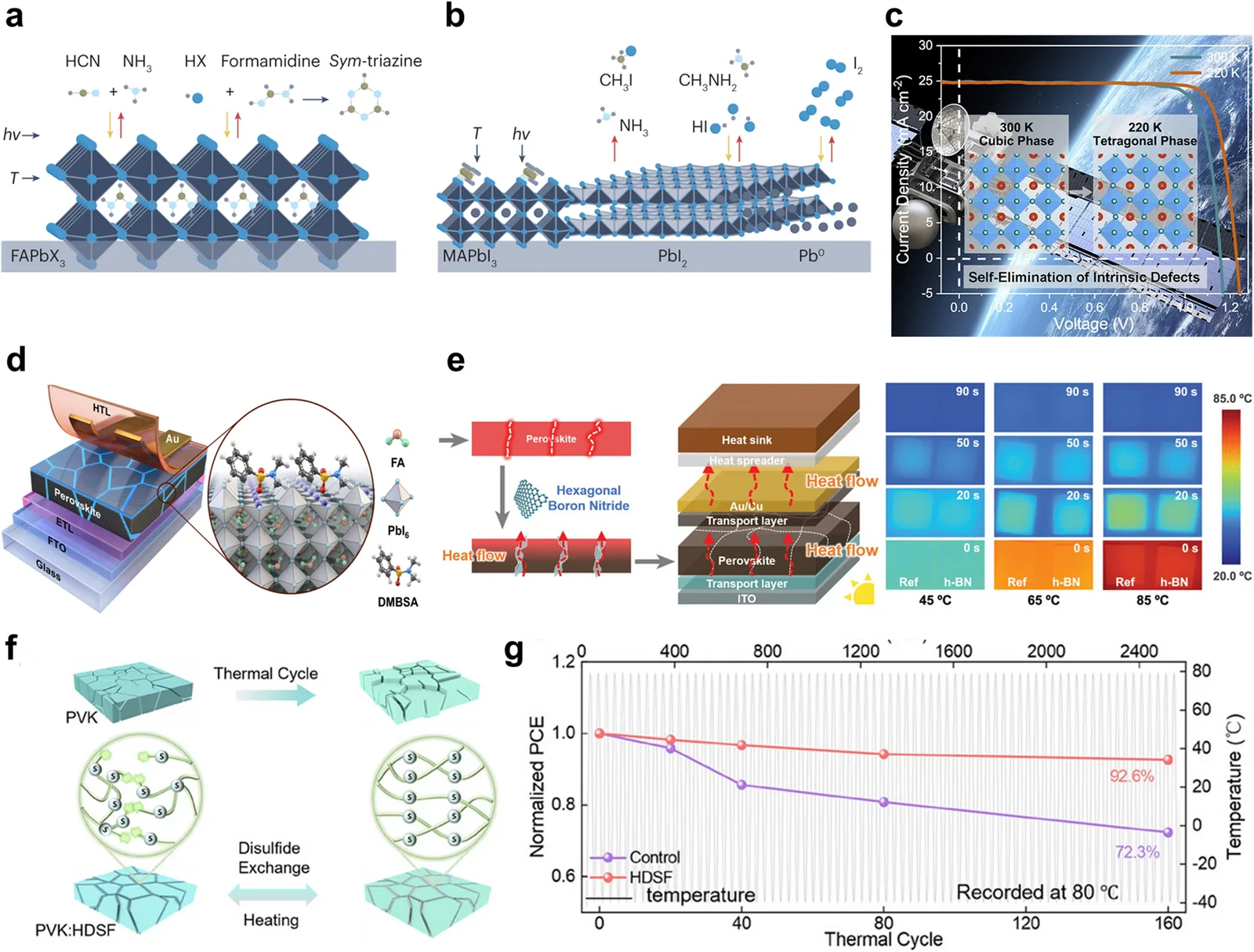
Thermally induced degradation of PSCs and corresponding mitigation strategies. **a** Degradation mechanisms of FAPbX_3_ under illumination and thermal stress. Red arrows indicate bond-breaking processes; yellow arrows indicate reforming processes. Irreversible pathways are denoted by single red arrows [14]. Copyright 2023, Springer Nature. **b** Degradation pathways of MAPbI_3_ under illumination and thermal conditions, following the same arrow conventions. **c** Photovoltaic performance of an optimized FAMACs-based PSC measured at 300 K and 220 K [81]. Copyright 2020, Elsevier. **d** Schematic of a PSC incorporating DMBSA for defect passivation within the perovskite layer [142]. Copyright 2025, John Wiley & Sons. **e** Schematic diagram of PSCs with enhanced heat dissipation and corresponding IR thermal images during a cooling test at various temperatures [145]. Copyright 2022, John Wiley & Sons. **f** Schematic illustration of heat-triggered self-healing behavior via dynamic disulfide bond exchange in HDSF [148]. Copyright 2025, John Wiley & Sons. **g** Normalized PCE evolution of the HDSF-based PSC during thermal cycling

The thermal stability of FAPbI_3_ is generally superior to that of MAPbI_3_. For instance, FAPbI_3_ films remain stable without visible discoloration after being held at 150 °C for 60 min, whereas MAPbI_3_ exhibits noticeable degradation after approximately 30 min [133]. This enhanced stability is attributed to the stronger interaction between FA^+^ and [PbI_6_]^4−^ than between MA + and [PbI_6_]^4−^ [134]. Nevertheless, FAPbI_3_ undergoes thermal decomposition at relatively low temperatures (> 50 °C), as formamidinium iodide (FAI) dissociates into FA and HI [134]. Both reactions are reversible and, in well-encapsulated devices, may not significantly impair performance. However, at around 95 °C, FA undergoes an irreversible conversion to *sym*-triazine and NH_3_ [135]. When the temperature exceeds the α-FAPbI_3_ phase-transition threshold (~ 160 °C), FAI volatilizes as HCN and NH_3,_ and partial decomposition of FAPbI_3_ into PbI_2_ occurs [136]. Figure 6a, b illustrates the degradation processes of FAPbI_3_ and MAPbI_3_ under thermal stress, respectively [14].

In contrast, CsPbI_3_ does not undergo low-temperature decomposition associated with organic loss, as it lacks volatile organic A-site species. The photoactive α-CsPbI_3_ phase remains stable up to approximately 390 °C; beyond this point, PbI_2_ volatilizes, leaving CsI as the residue [137]. Accordingly, all-inorganic perovskites may offer advantages in extreme environments that demand high-temperature operation.

On the other hand, the applicability of PSCs at low temperatures appears promising. In an early study, Ginting et al. reported that the PCE of PSCs increased from 14.2% to 15.5% as the temperature decreased from 298 to 253 K, primarily due to increases in *J*_SC_ and *V*_OC_, along with reduced hysteresis [138]. Similarly, Chen et al. observed substantial performance enhancement in (FA, MA, Cs)Pb(I, Br)_3_ PSCs over the 290–180 K range, with a peak PCE of 25.2% at 220 K compared to 23.3% at 300 K (Fig. 6c) [81]. However, when the temperature was further reduced to 130 K, the PCE declined to 17.1%, primarily due to a reduced FF, likely linked to low-temperature phase transitions in the perovskite. The authors proposed that the PCE enhancement at intermediate low temperatures arises from phase- or strain-induced lattice distortions that activate self-elimination of intrinsic defects, thereby improving *V*_OC_ (from 1.153 to 1.229 V) and overall efficiency. These findings suggest that PSCs may be particularly well suited for low-temperature applications beyond conventional terrestrial environments.

Moreover, during thermal cycling, mismatches in the CTEs among layers lead to highly non-uniform strain distributions within PSCs [139]. Rapid and periodic temperature fluctuations can accelerate mechanical fatigue and even result in device failure through delamination of the layered stack. Unlike fixed-temperature exposure, thermal cycling imposes repeated mechanical stress and dynamic strain due to the continual expansion and contraction of CTE-mismatched layers. However, accelerated aging studies of PSCs under thermal-cycling conditions remain relatively limited compared to those conducted at constant temperatures. Ensuring reliable operation in extreme environments (e.g., space), where rapid thermal cycling is prevalent, necessitates ongoing thermal-cycling qualification testing, as well as the implementation of design and materials strategies to enhance stability.

#### Approaches for Mitigating Thermal Degradation

Thermal degradation in PSCs is fundamentally associated with the low formation enthalpy of metal halide perovskites and the presence of volatile organic components, which can be released even at moderately elevated temperatures, thereby triggering irreversible material loss and device degradation [57]. Using gas chromatography–mass spectrometry (GC–MS), Shi et al. identified gaseous decomposition products generated upon heating perovskite films, including CH_3_I, *sym*-triazin (C_3_H_3_N_3_), CH_3_Br, and NH_3_ [140]. They further demonstrated that advanced encapsulation can effectively suppress thermal degradation. In particular, a polymer–glass blanket encapsulation provided a hermetic, pressure-tight environment that prevented the escape of volatile degradation products. Thus, advanced encapsulation strategies play a critical role in mitigating thermally induced degradation.

Ammonium-based interfacial modifiers, widely used in high-performance PSCs, can enhance initial PCE but often compromise thermal stability. At elevated temperatures, the high pKa of these modifiers promotes deprotonation, which subsequently triggers adverse reactions with perovskite components, accelerating material degradation [141]. To address this issue, Kang et al. employed the non-ionic passivation agent *N,N*-dimethylbenzenesulfonamide (DMBSA), which offers both a low pKa and effective defect passivation (Fig. 6d) [142]. Compared with ionic PEA^+^, DMBSA-based PSCs exhibited significantly enhanced thermal stability: After 1500 h at 85 °C, DMBSA-passivated devices retained 96.1% ± 0.8% of their initial PCE, whereas PEA^+^-passivated devices retained only 64.0% ± 0.19%.

Enhancing heat transfer within the device provides a complementary strategy. Incorporation of high-thermal-conductivity materials such as Al_2_O_3_, SiO_2_, or hexagonal boron nitride (h-BN) can facilitate thermal dissipation [143–146]. Yang et al. identified the perovskite layer as the primary source of waste heat under illumination [145]. To mitigate this, they strategically placed h-BN at grain boundaries and on the film surface, and coupled the device stack to an external copper heat sink (Fig. 6e). This approach reduced the operating temperature by approximately 6.5 °C under AM 1.5G illumination and significantly improved long-term stability: Optimized devices retained 96% of their initial PCE after 1704 h at 85 °C and 92% after 2164 h of maximum-power-point tracking.

The stability of PSCs under thermal cycling remains a critical consideration. During thermal cycling, the CTE mismatch between the perovskite and adjacent layers induces layer-dependent contraction and expansion rates, particularly at interfaces [139]. This mismatch promotes mechanical delamination and accelerates device failure. These stresses can be mitigated by balancing compressive and tensile forces across the stack; for instance, employing a polymer substrate with a CTE more closely matched to that of the perovskite can reduce mismatch-induced stress [147]. Additionally, interfacial modification can enhance thermal tolerance. Li et al. incorporated β-poly(1,1-difluoroethylene) into PSCs and subjected the devices to 120 rapid thermal cycles between − 60 and + 80 °C at 20 °C min^−1^ [82]. After cycling, control devices retained 75.6% of their initial efficiency at + 80 °C and 63.0% at − 60 °C, while the modified devices preserved 93.9% and 88.7%, respectively. More recently, Tang et al. implemented a thermally activated dynamic self-healing framework (HDSF) to repair grain-boundary defects induced by thermal fluctuations, thereby improving temperature stability [148]. The HDSF was synthesized via dehydration and polycondensation of 4-aminophenyl disulfide (APD) with tris(4-formylphenyl)amine (TPA), forming a dynamic covalent network (Fig. 6f). Under variable-temperature operation, the HDSF regulates thermomechanical stress and heals thermally induced grain-boundary defects through the dynamic exchange of disulfide bonds. Even after 160 rapid thermal cycles between − 60 °C and + 80 °C at 20 °C min^−1^, HDSF-integrated devices retained 87.6% of their initial efficiency at − 40 °C and 92.6% at + 80 °C (Fig. 6g). This thermally driven dynamic self-healing strategy significantly enhances the reliability of PSCs for extreme-environment applications.

To enable the deployment of PSCs in extreme environments, thermal stability must be engineered at the material, interface, stack, and packaging levels, as summarized in Table 3. Key strategies include advanced encapsulation to suppress the loss of volatile by-products, enhanced heat dissipation using high-thermal-conductivity materials and external heat sinks, and reduced CTE mismatch through CTE-aware layer selection and compatible interlayers. Self-healing approaches that repair thermally induced degradation also represent a promising complementary solution. Future advancements will require standardized thermal-cycling qualification protocols, multistress testing that simultaneously incorporates heat, humidity, illumination, bias, vacuum, and radiation, and system-level designs that retain the volumetric and power-conversion advantages of PSCs while ensuring long-term operational reliability.

**Table 3:** Representative thermal management strategies for improving stability of PSCs

Thermal management strategies	PCE (%)	Measurement conditions	Stability	References
Advanced encapsulation	~ 19%	85°C, 85% RH	> 95% PCE retention after 1800 h	[140]
Thermally stable passivation (*N*,*N*-dimethylbenzenesulfonamide)	25.4	85°C, N_2_	> 96% PCE retention after 1500 h	[142]
Heat dissipation via h-BN (GB/surface) + external Cu heat sink	22.8	85°C, N_2_	> 96% PCE retention after 1704 h	[145]
Heat dissipation via multiwalled carbon nanotubes within perovskite	23.1	85°C, 35% RH	> 89% PCE retention after 1300 h	[149]
Inorganic HTL (Co_X_S_Y_)	24.4	85°C, N_2_	> 90% PCE retention after 1000 h	[150]
Dopant-free HTL	25.6	85°C, 85% RH	> 85% PCE retention after 1000 h	[151]
Reduce CTE-mismatch stress via interfacial modifier (β-poly(1,1-difluoroethylene))	24.6	Rapid thermal cycles (− 60 to + 80 °C, 20 °C min^−1^)	PCE retention after 120 thermal cycles: 93.9% (+ 80 °C), 88.7% (− 60 °C)	[82]
Thermally activated dynamic self-healing framework	26.3	Rapid thermal cycles (− 60 to + 80 °C, 20 °C min^−1^)	PCE retention after 160 thermal cycles: 92.6% (+ 80 °C), 87.6% (− 40 °C)	[148]

### Water

#### Degradation Pathways of PSCs Under Humid Conditions

**Fig. 7:**
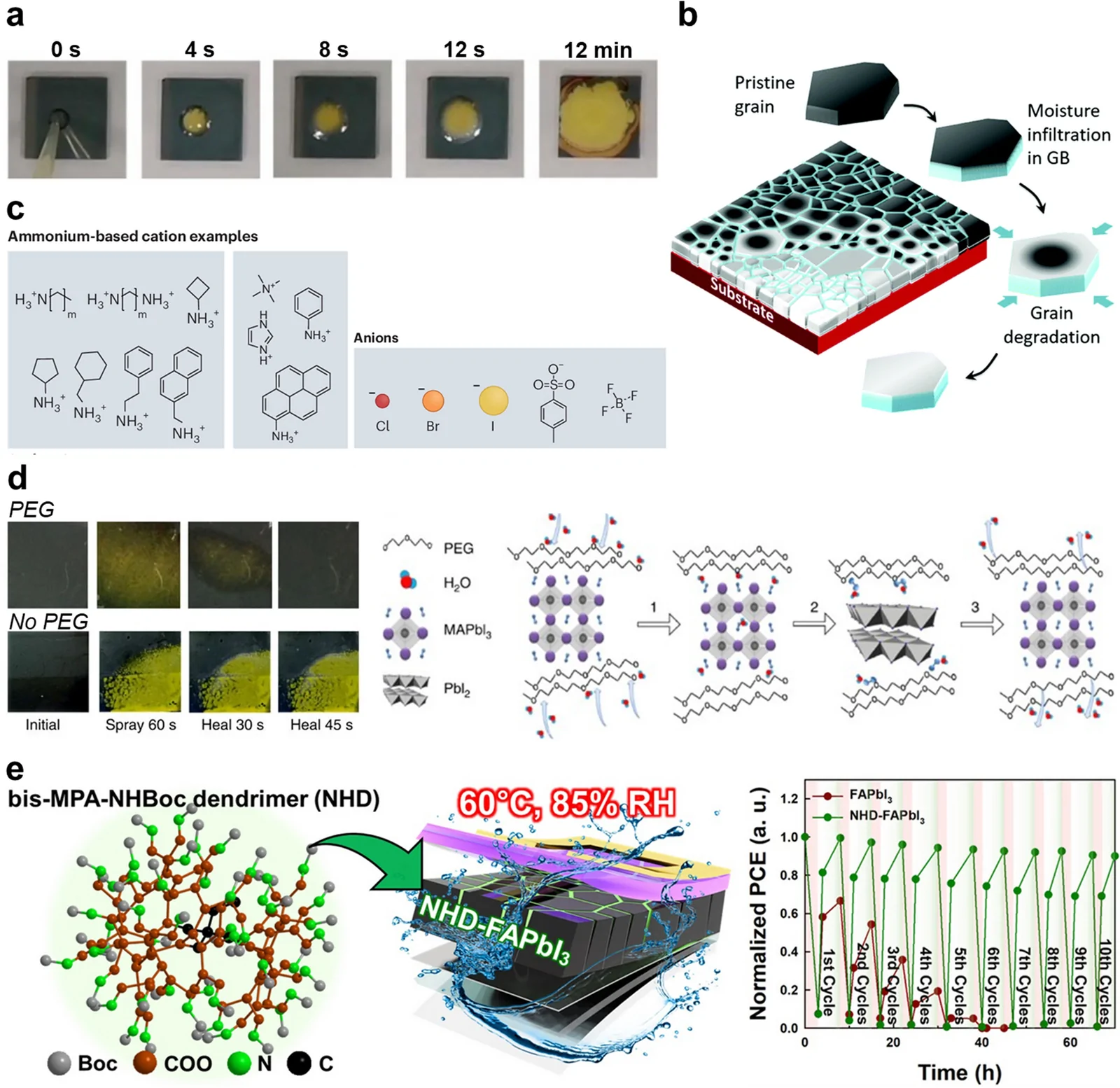
Moisture-induced degradation mechanisms in PSCs and corresponding mitigation strategies. **a** Photographs showing the degradation of a perovskite film over time after water droplet exposure [152]. Copyright 2022, Elsevier. **b** Schematic illustration of moisture-induced degradation in perovskite films, where degradation initiates at specific grain boundaries and propagates laterally across the film [155]. Copyright 2017, RSC Publishing. **c** Representative ammonium-based cations and anions used in salt additives for defect passivation at the surface and interface of 3D perovskite layers [19]. Copyright 2024, Springer Nature. **d** Photographs comparing perovskite films with and without PEG, showing color change after 60 s of water spraying and 45 s of ambient air exposure; accompanying schematic shows the self-healing mechanism in PSCs [168]. Copyright 2016, Springer Nature. **e** Molecular structure of the fourth-generation bis-MPA-NHBoc dendrimer (NHD) and a schematic of the NHD-based PSC; recovery cycling tests comparing normalized PCE of pristine and NHD-incorporated PSCs [56]. Copyright 2025, John Wiley and Sons

Perovskites exhibit intrinsic instability and are highly sensitive to moisture, as demonstrated by their rapid color change from black (photoactive) to yellow (non-photoactive) within minutes under humid conditions (Fig. 7a) [152]. Numerous studies have shown that atmospheric moisture interacts with perovskites through various pathways, accelerating degradation and undermining chemical stability. Walsh et al. proposed a simple acid–base reversible reaction between MAPbI_3_ and H_2_O, with a possible degradation mechanism outlined in Eqs. (14) and (15) [153]:

14 $$ \left[ {\left( {{\mathrm{CH}}_{3} {\mathrm{NH}}_{3} } \right){\mathrm{PbI}}_{3} } \right] + {\mathrm{H}}_{2} {\mathrm{O}}\, \rightleftarrows \,\left[ {\left( {{\mathrm{CH}}_{3} {\mathrm{NH}}_{3} } \right)_{n - 1} \left( {{\mathrm{PbI}}_{3} } \right)_{n} } \right]\left[ {{\mathrm{H}}_{3} {\mathrm{O}}^{ + } } \right] + {\mathrm{CH}}_{3} {\mathrm{NH}}_{2} \uparrow $$

15 $$ \left[ {\left( {{\mathrm{CH}}_{3} {\mathrm{NH}}_{3} } \right)_{n - 1} \left( {{\mathrm{PbI}}_{3} } \right)_{n} } \right]\left[ {{\mathrm{H}}_{3} {\mathrm{O}}^{ + } } \right]\, \rightleftarrows \, \left[ {\left( {{\mathrm{CH}}_{3} {\mathrm{NH}}_{3} } \right){\mathrm{PbI}}_{3} } \right]_{{\left( {n - 1} \right)}} + {\mathrm{PbI}}_{2} + {\mathrm{H}}_{2} {\mathrm{O}} + {\mathrm{HI}} \uparrow $$

By combining Eqs. (14) and (15), the overall decomposition process of MAPbI_3_ in the presence of moisture can be summarized as Eq. (16):

16 $$ \left[ {\left( {{\mathrm{CH}}_{3} {\mathrm{NH}}_{3} } \right){\mathrm{PbI}}_{3} } \right] + {\mathrm{H}}_{2} {\mathrm{O}}\, \rightleftarrows \, \left[ {\left( {{\mathrm{CH}}_{3} {\mathrm{NH}}_{3} } \right){\mathrm{PbI}}_{3} } \right]_{{\left( {n - 1} \right)}} + {\mathrm{PbI}}_{2} + {\mathrm{CH}}_{3} {\mathrm{NH}}_{2} \uparrow + {\mathrm{HI}} \uparrow $$

During this process, CH_3_NH_2_ and HI, which are gaseous at room temperature, are continuously released, thereby driving the reaction forward. Consequently, MAPbI_3_ ultimately decomposes into PbI_2_ when exposed to moisture in an open system [154].

#### Approaches for Mitigating Moisture-Induced Degradation

In commercial devices, robust encapsulation can render moisture a less dominant stressor compared to thermal or photochemical loads [14, 19]. Nevertheless, effective moisture management remains essential for long-term reliability, as slow permeation over multiyear operational lifetimes can induce perovskite decomposition and hydrate formation. Perovskite films are typically polycrystalline, and moisture-induced degradation predominantly initiates at exposed surfaces and grain boundaries (GBs) (Fig. 7b) [155]. Therefore, protecting these regions is critical to ensuring device durability.

An effective materials-level approach for improving perovskite stability involves engineering 2D/3D perovskite heterostructures in which low-dimensional perovskite phases incorporating bulky organic spacer cations form at the surface of conventional 3D absorbers. These surface-localized 2D phases introduce hydrophobic organic components that suppress moisture ingress while modifying interfacial electronic structures in a manner favorable for charge extraction [156–158]. As a result, representative 2D/3D PSCs have been reported to exhibit minimal performance degradation even after prolonged outdoor exposure exceeding 800 h [157–159]. These results indicate that 2D/3D heterostructure engineering can complement encapsulation and passivation strategies against moisture- and water ingress-driven degradation in humid extreme environments.

Practical strategies include surface passivation using hydrophobic, bulky ammonium cations (R − NH_3_^+^, where R = *n*-butyl, isobutyl, *n*-hexyl, *n*-octyl, oleyl, phenethyl, etc.) paired with counter-anions such as halides (I^−^, Br^−^, Cl^−^), sulfate (SO_4_^2−^), phosphate (PO_4_^3−^), trifluoroacetate (CF_3_COO^−^), or tosylate (TsO^−^) to passivate the perovskite surface (Fig. 7c) [19, 160–164]. These methods involve depositing dissolved salts onto the surface. Grain boundaries can be passivated using similar molecules introduced directly into the perovskite precursor; during crystallization, they localize at GBs and protect moisture-sensitive sites. Polymer additives can serve a similar function. For instance, incorporating 1–3 mol% poly(vinyl alcohol) achieved > 720 h of stability at 90% RH in unencapsulated PSCs [162]. Moisture management can also extend to the surrounding charge-transport layers [165–167]. For example, Koo et al. employed Co_*x*_S_*y*_ nanoparticles capped with organic ligands as the hole-transport layer (HTL) [150]. Under conditions of 65 °C and 65% RH, devices using hygroscopic Spiro-OMeTAD as the HTL showed rapid performance degradation, whereas those with the Co_*x*_S_*y*_-based HTL retained ~ 95% of their initial PCE after 1000 h.

Another strategy to enhance moisture stability is to impart self-healing functionality to the perovskite layer by leveraging the intrinsic reversibility of moisture-induced decomposition. In this approach, volatile species generated under humid conditions are retained near the film and, upon drying, are reabsorbed to drive reformation of the perovskite phase, thereby restoring both structure and performance. Zhao et al. employed a polyethylene glycol (PEG)-based polymer scaffold to inhibit the volatilization of MA in MAPbI_3_ perovskite [168]. Embedding the PEG scaffold within the perovskite layer introduced hydrogen-bonding interactions between PEG and volatile MA, effectively anchoring MA in proximity to the scaffold (Fig. 7d). Upon natural drying, the retained MA molecules reacted with PbI_2_, enabling recrystallization of the MAPbI_3_ phase and leading to the recovery of PSC performance.

In addition to this approach, we recently proposed dendrimers as more efficient absorbents for volatile species [56]. Dendrimers feature a highly organized, hyperbranched, tree-like architecture that, compared with linear polymers such as PEG, provides a significantly higher density of functional groups per unit volume as well as internal cavities that serve as molecular reservoirs. These cavities can capture and retain volatile components through specific interactions during moisture-induced degradation. In this study, FAPbI_3_-based PSCs incorporating the dendrimer retained ~ 90% of their initial PCE after ten degradation–recovery cycles, with degradation conducted at 60 °C and 85% RH, and recovery at 25 °C and < 20% RH (Fig. 7e). Consistent with diffusivity differences (H_2_O > FA), water evaporated first during recovery, followed by the release of FA, which facilitated self-healing. Furthermore, the dendrimer suppressed the moisture-driven, irreversible degradation pathway leading to oxidized Pb formation under high humidity, thereby enabling repeated and sustainable self-healing in the devices.

Overall, achieving durable PSCs requires an integrated strategy that combines robust encapsulation with hydrophobic surface and grain-boundary protection, moisture-tolerant charge-transport layers, and self-healing capability. Such integration is essential for stability in extreme environments, including aqueous conditions, where permeation and corrosion are significantly intensified. Representative moisture management strategies for improving the stability of PSCs are summarized in Table 4.

**Table 4:** Representative moisture management strategies for improving stability of PSCs

Moisture management strategies	PCE (%)	Measurement conditions	Stability	References
2D capping layer via oleylammonium iodide + encapsulation	24.3	85°C, 85% RH	> 95% PCE retention after 1000 h	[169]
Polymer (PMMA) capping layer	16.3	50 ~ 70% RH	> 91% PCE retention after 50 day	[170]
Polymer (PVA) additive	17.4	90% RH	> 90% PCE retention after 30 day	[162]
Moisture-tolerant HTL (ligand-capped Co_X_S_Y_ nanoparticle)	24.4	65°C, 65% RH	> 95% PCE retention after 1000 h	[150]
Self-healing via volatile retention (polymer scaffold)	~ 16%	70% RH	> 65% PCE retention after 300 h	[168]
Self-healing via volatile reservoir (dendrimer)	26.2	Degrade: 60 °C, 85% RH; Recover: 25 °C, < 20% RH	> 90% PCE retention after 10 degradation–recovery cycles	[56]
Underwater durability via advanced encapsulation (PIB edge sealing)	7.5	3.5 wt% NaCl, immersion	Pb release within stringent drinking-water limits after 10 days	[171]

### Mechanical Stress

In extreme environments, mechanical stability can be a key factor in determining PSC reliability. Considering that PSCs consist of multiple layers of thin films, including a perovskite absorber, charge-transport layers (CTLs), and electrodes, several components are mechanically fragile and/or exhibit limited interfacial adhesion. As a result, pressure fluctuations, vibration/impact, and repeated deformation can readily initiate cracking and delamination. Importantly, strain is typically heterogeneous in layered thin-film stacks and tends to localize near microstructural and geometrical discontinuities (e.g., grain boundaries, interfacial roughness/heterogeneity, thickness steps, edges, and preexisting defects) [172–174]. Therefore, damage primarily initiates at these weak points and grows in a fatigue manner under repeated loading. Electrical performance degradation can occur directly due to increased series resistance from electrode cracking, loss of electrical contact from interface debonding, and the formation of local shunt paths from crack penetration. Accordingly, improving mechanical stability should focus more on designing the device stack to suppress crack initiation, slow crack propagation, and resist interfacial failure rather than simply increasing strength.

As a first step, enhancing the mechanical robustness of the perovskite absorber is important. Notably, halide perovskites have been reported to be mechanically softer than conventional photovoltaic semiconductors such as Si and III–V compounds due to their ionic bonding character, which can help mitigate stress concentration in thin films [175]. In addition, partial crack-healing behavior has been reported under mild thermal annealing (e.g., ~ 80 °C) or under compressive stress [176, 177]. This behavior can be described by the soft crystalline lattice, low energy of formation, low activation energy for ionic migration, and the dynamic nature of the defects. The combination of these features can facilitate ion diffusion and allow the crystals to repair after mechanical damage.

In addition to these intrinsic features, recent studies have focused on improving mechanical durability by reinforcing microstructural weak points, particularly grain boundaries where cracks preferentially initiate [178]. Polymer additives or polymerizable components can be preferentially localized at grain boundaries, where they form cross-linked polymer networks. These networks redistribute stress and increase energy dissipation, improving resistance to cracking. For example, Li et al. introduced a dopamine-functionalized three-dimensional hyperbranched polymer that promotes multidentate bonding and cross-linking at perovskite grain boundaries [179]. This strategy strengthened bonding within the film, and flexible PSCs maintained 94.1% of their initial PCE after 10,000 bending cycles at a 3 mm radius under 65% relative humidity.

Mechanical failure frequently occurs not only within the perovskite layer but also as delamination at interfaces such as ETL/perovskite or HTL/perovskite. Interfacial fracture can directly interrupt charge-transport pathways and increase contact resistance. Therefore, device designs that raise interfacial fracture energy (adhesion toughness) and distribute shear deformation at critical interfaces are important under extreme conditions including repeated pressure changes, bending, vibration, and thermomechanical cycling. In this regard, Dai et al. showed that self-assembled monolayers (SAMs) can increase interfacial adhesion toughness at perovskite interfaces, suggesting that interfacial engineering can enhance mechanical delamination resistance [180]. Furthermore, Li et al. focused on the intrinsically low interfacial fracture energy between the ETL/perovskite, which makes the interface susceptible to delamination under mechanical loading [181]. They showed that introducing a multidentate hyperbranched polymer can strengthen interfacial bonding and improve delamination resistance.

Moreover, mechanically flexible electrodes and CTLs are required to maintain charge collection capability under deformation. Brittle transparent conducting oxides (e.g., ITO) and rigid inorganic CTLs can act as crack initiation sites, and progressive microcracks can increase resistance and accelerate performance loss under repeated loading. Network-type conductors, including metal grids/meshes, Ag nanowire (AgNW) networks, and hybrid network electrodes, have been widely considered as alternatives or complements to ITO [182–184]. They can preserve conduction pathways even when local cracks form, thereby mitigating fatigue-induced resistance increases. For instance, Im et al. reported that AgNW-based metal network electrodes enabled flexible PSCs to retain conductive pathways after bending, alleviating the crack-driven performance degradation commonly observed for ITO electrodes [185].

Overall, mechanical reliability in extreme environments is governed by how effectively the device stack blocks the failure pathway from crack initiation to fatigue growth and interfacial fracture. Strengthening the perovskite absorber layer, enhancing interfacial adhesion and stress redistribution, and incorporating mechanically flexible electrodes and CTLs are practical approaches to improve the durability of PSCs in scenarios where mechanical loading is unavoidable. Representative mechanical stress management strategies for enhancing PSC stability are summarized in Table 5.

**Table 5:** Representative mechanical stress management strategies for improving stability of PSCs

Mechanical stress management strategies	PCE (%)	Measurement conditions	Stability	References
GB reinforcement via multidentate cross-linking hyperbranched polymer	24.4	65% RH, bending radius 3 mm, 10,000 cycles	Flexible PSC retains 94.1% initial PCE	[179]
Increase interfacial adhesion toughness using SAMs	21.4	LED, N_2_	> 80% PCE retention after 4000 h	[180]
Interfacial bonding via multidentate hyperbranched polymer at ETL/perovskite	23.9	bending radius 3 mm, 10,000 cycles	Flexible PSC retains 88.9% initial PCE	[181]
Network electrodes (ITO/Ag NW-GFRHybrimer)	12.1	bending radius 2.5 mm, 500 cycles	Flexible PSC retains ~ 90% initial PCE	[185]
Network electrodes (ITO/Cu NW-GFRHybrimer)	13.0	bending radius 2.5 mm, 500 cycles	Flexible PSC retains ~ 80% initial PCE	[185]

### Encapsulation

Intrinsic stability enhancement strategies are essential for mitigating thermodynamic and chemical instabilities in the perovskite device stack. Nevertheless, intrinsic approaches alone are typically insufficient to ensure durable operation in real environments. In practical deployment, extrinsic stressors such as moisture and oxygen ingress tend to dominate performance loss. Under these conditions, encapsulation becomes the primary means of limiting environmental exposure and maintaining device performance. Accordingly, we discuss encapsulation strategies for PSCs operating in extreme environments, focusing on stressor-specific encapsulation architectures and materials.

PSC encapsulation can be broadly classified into two architectures: (i) glass–glass encapsulation and (ii) thin-film encapsulation (TFE). Glass–glass encapsulation employs front and rear glass covers, a lamination encapsulant (adhesive), and an edge seal, offering strong barrier performance and mechanical protection. In commercial silicon photovoltaics, lamination adhesives such as ethylene–vinyl acetate copolymer (EVA), silicones, and polyvinyl butyral (PVB) are widely adopted to achieve durable module packaging. Similarly, PSCs can be sandwiched between two glass plates and sealed with thermoplastic or thermoset sealants, or with UV-curable epoxy/resin systems (desiccants can be incorporated to mitigate residual moisture as needed). In contrast, TFE suppresses moisture/oxygen transmission by depositing barrier stacks directly on the device surface, which is attractive for ultralight and flexible implementations. However, the operational lifetime of TFE is highly sensitive to pinhole defects and the integrity of edge and termination sealing. Consistent with experience in thin-film PV technologies such as GaAs and Cu(In,Ga)Se_2_ (CIGS), a broad range of organic and inorganic encapsulation materials has been explored for PSCs [186–188]. Common organic layers include poly (methyl methacrylate) (PMMA), polyethylene terephthalate (PET), polytetrafluoroethylene (PTFE), polycarbonate (PC), polydimethylsiloxane (PDMS), whereas inorganic layers typically include Al_2_O_3_, SiO_*X*_, TiO_2_, SiN_*X*_. These materials and their trade-offs (barrier performance, mass, flexibility, and process complexity) have been systematically summarized in PSC encapsulation reviews and experimental studies [186–188].

In extreme environments, barrier performance becomes even more critical, and encapsulation requirements should be further specified according to the dominant stressors in each scenario. However, systematic comparisons and validations of encapsulation materials, processes, and architectures under extreme-environment conditions remain limited, and many reports are still confined to individual proof-of-concept studies. Space is a representative case because it involves high-energy particle radiation and AO, both of which can erode and degrade organic materials. Accordingly, encapsulation approaches developed for terrestrial conditions may not be sufficient. Seid et al. reported that after AO exposure (2 h), the average PCE of unencapsulated PSCs decreased to ~ 40% of the initial value, whereas devices protected by a ~ 700 nm SiO barrier retained more than 97% of the initial PCE [189]. They further showed that PEAI-based 2D passivation alone did not fundamentally suppress AO-induced degradation, highlighting the critical role of inorganic AO-blocking barriers. In radiation environments, space-grade coverglass is a standard packaging choice. Prior studies indicate that coverglass can partially mitigate electron/proton-induced damage and that radiation-resistant designs (e.g., Ce-doped coverglass) can reduce radiation-induced darkening of the glass itself, thereby limiting optical transmission losses [190–192].

Large temperature fluctuations encountered in deserts, polar regions, and space impose additional thermomechanical constraints. Accumulated stresses arising from CTE mismatches can lead to delamination and cracking. Therefore, encapsulation should be optimized not only for barrier properties but also for mechanical compliance, adhesion retention, and process conditions. In this context, low-stress packaging strategies employing viscoelastic adhesive interlayers and/or stress-buffer layers can be reasonable approach to improve thermal-cycling reliability [193].

Underwater operation involves continuous exposure to liquid water, where even minor leakage can cause rapid and severe performance loss. Therefore, key requirements include long-term seal integrity under immersion, water-resistant adhesion, and chemical stability against salinity. In addition, because environmental safety is directly coupled to deployment in water, encapsulation should also be designed to prevent lead leakage rather than relying solely on barrier function. For example, phosphonic acid (–PO_3_H_2_) and sulfonic acid (R–SO_3_H) functionalities can act as strong Pb^2+^ sorbents, and integrating such sorbents into encapsulation components has been proposed to suppress lead leakage [194]. Li et al. implemented a transparent Pb-absorbing *P,P′*-di(2-ethylhexyl)methanediphosphonic acid (DMDP) film on the glass side, and on the metal electrode side, an encapsulation film containing poly(ethylene oxide) (PEO) and Pb-chelating agent *N,N,N′,N′*-ethylenediaminetetrakis(methylenephosphonic acid) (EDTMP) [195]. This strategy achieved more than 96% Pb adsorption even when damaged PSCs were immersed in an acidic rainwater environment (pH 4.2). Underwater deployment also requires addressing optical losses caused by surface contamination and biofouling, which reduce transmittance and long-term power output. Antifouling approaches for PSCs are not yet widely developed, but they are relevant for practical underwater use. For example, a transparent antifouling coating based on Cu_2_O/ZnO nanoparticles, an organic booster biocide, and a fast-polishing binder maintained high visible-light transmittance for up to three months in tropical seawater without mechanical cleaning [196].

In many extreme-environment applications, the risk of unexpected damage cannot be fully eliminated. Therefore, encapsulation strategies that account for post-damage scenarios are also important. One representative approach is self-healing polymer encapsulation, which relies on polymers that can repair cracks when heated above the glass-transition temperature (*T*_g_). Jiang et al. encapsulated PSCs with a self-healing epoxy resin (ER), induced damage using a hail impact protocol (modified FM 44787 standard), and evaluated Pb leakage [197]. The ER has a *T*_g_ of 42 °C, which is attainable under sunlight exposure. Above *T*_g_, microcracks healed and hydrophobicity was recovered, decreasing the Pb leakage rate from 30 to 0.08 mg h^−1^ m^−2^.

In summary, a single universal encapsulation solution is unlikely to meet the requirements for PSC deployment in extreme environments. Instead, advanced and scenario-specific encapsulation strategies are required for space (AO, radiation, and vacuum), deserts and polar regions (thermal cycling and mechanical fatigue), and underwater operation (long-term immersion, salinity, and biofouling). However, systematic validation and comparative studies of encapsulation materials and architectures under environment-relevant conditions remain limited. In particular, for space and mobile platforms, encapsulation should be designed not only for barrier performance but also with the mass penalty and its impact on specific power (W kg^−1^) in mind. In the future, standardized test protocols will be needed to evaluate both performance retention and safety under representative stressors for each target environment.

Table 6 summarizes the major environmental stress factors, associated degradation mechanisms, representative degradation behaviors, and corresponding mitigation strategies in PSCs.

**Table 6:** Summary of major environmental stress factors, underlying degradation mechanisms, representative degradation behaviors, and corresponding mitigation strategies in PSCs

Stress factor	Degradation mechanisms	Representative degradation behaviors	Mitigation strategies
Light	Defect-mediated ion migration and trap activation, UV-driven decomposition and chain reactions (e.g., PbI_2_ → Pb^0^ + I_2_), accelerated photooxidation pathways, photocatalytic transport layers (e.g., TiO_2_) promoting iodide oxidation/Pb^2+^ reduction, photoassisted volatile loss	*V*_OC_ loss (increased non-radiative recombination) and FF drop (transport barriers/contact deterioration), hysteresis increase and MPP drift (photoassisted ion migration/metastable trap accumulation), emergence of Pb^0^ signatures and optical bleaching/discoloration. For mixed-halides, phase segregation manifested as PL/EL peak splitting or spectral shift and corresponding *J*–*V* instability	Defect passivation to suppress ion migration, UV management (UV filters, UV-blocking cover layers), replacing/engineering photocatalytic CTLs, self-healing concepts coupled with volatile retention
Thermal	Thermally induced volatilization of organic species, low formation enthalpy enabling decomposition at moderate T, thermally activated ion migration/interdiffusion, thermal-cycling-induced fatigue from CTE mismatch across layers	Under constant heat: progressive FF decrease with *R*s increase (contact degradation/interdiffusion) and *V*_OC_ decrease (interfacial recombination), absorption loss/film yellowing (PbI_2_ formation) and electrode/CTL degradation at elevated T, under thermal cycling: Step-like PCE drops after certain cycles, consistent with microcracking/delamination, sometimes accompanied by *R*sh decrease (incipient shunts) and abrupt failure	Thermally stable passivation/interfacial modifiers, heat dissipation (high-thermal-conductivity fillers, heat sinks), CTE-aware stack design and compatible interlayers, dynamic self-healing frameworks for cycling-induced defects
Moisture	Hydration-assisted decomposition initiating at surfaces/GBs, volatile release, moisture-assisted corrosion/interfacial degradation, slow permeation over long lifetimes	Early stage: hysteresis increases and *V*_OC_ loss (GB/surface hydration and trap activation), progression: FF loss with rising *R*s (transport-layer/electrode corrosion, interfacial deterioration), advanced stage: Rapid PCE collapse once water reaches interfaces/contacts, observable color change (photoactive → non-photoactive)	Robust encapsulation and edge sealing, hydrophobic surface/GB passivation (bulky ammonium salts, polymers), moisture-tolerant CTLs, reversible/self-healing concepts that retain volatiles locally
Mechanical	Strain localization at discontinuities (GBs, heterogeneous interfaces, thickness steps, edges, preexisting defects) → crack initiation, fatigue crack growth, interfacial delamination due to low adhesion toughness, brittle electrodes/CTL microcracking	Cyclic loading/bending: FF loss via *R*s increase (electrode/CTL microcracks and disrupted current collection), severe cases: *R*sh decrease and sudden *V*_OC_/FF collapse when cracks penetrate to form shunts, stepwise degradation after a threshold number of cycles consistent with fatigue growth, interfacial delamination leads to contact loss and local performance non-uniformity	Absorber toughening (polymer additives or polymerizable components localized at GBs), interfacial toughening (e.g., SAM-enabled adhesion-toughness improvements), mechanically compliant conductors (e.g., metal grids/meshes, Ag nanowires, hybrid networks)

## PSCs under Real Extreme Conditions

Field demonstrations under outdoor and other extreme conditions are steadily increasing to evaluate the practical applicability of PSCs. Devices that pass damp-heat testing (85 °C/85% RH, 1000 h) and maintain strong performance in outdoor environments demonstrate growing maturity in encapsulation strategies and device architecture [169, 187]. In aquatic or saline environments, studies have examined shallow-water operation, encapsulant corrosion resistance, and Pb leaching during seawater immersion [59, 62]. Some reports indicate that Pb release remains within stringent water-quality limits even after 10 days of saline immersion in damaged devices [171]. Degradation mechanisms arising from combined stressors—such as radiation, AO, vacuum, and rapid thermal cycling—in space and high-altitude environments are being elucidated through both flight missions and simulation tests [10, 30, 192, 198]. Overall, ensuring high durability in extreme environments requires qualification under multiple simultaneous stress conditions, including thermal, humidity, light, salinity, and pressure. This section discusses reported field implementations of PSCs under such extreme conditions.

### Space

The first space experiment on PSCs was reported by Cardinaletti et al. who mounted MAPbI_3_ PSCs on a balloon launched from the Esrange Space Center (northern Sweden) in October 2016 [199]. The flight lasted approximately 5 h, with more than 3 h spent in the stratosphere, reaching an altitude of ~ 32 km—roughly three times that of typical commercial flights. Device performance was retained without complete degradation during the flight; the PCE decreased from 14.6% to 9.3%. The authors emphasized the need for improved encapsulation, testing under extreme thermal cycling, and strategies to mitigate pressure-induced performance changes for space applications.

**Fig. 8:**
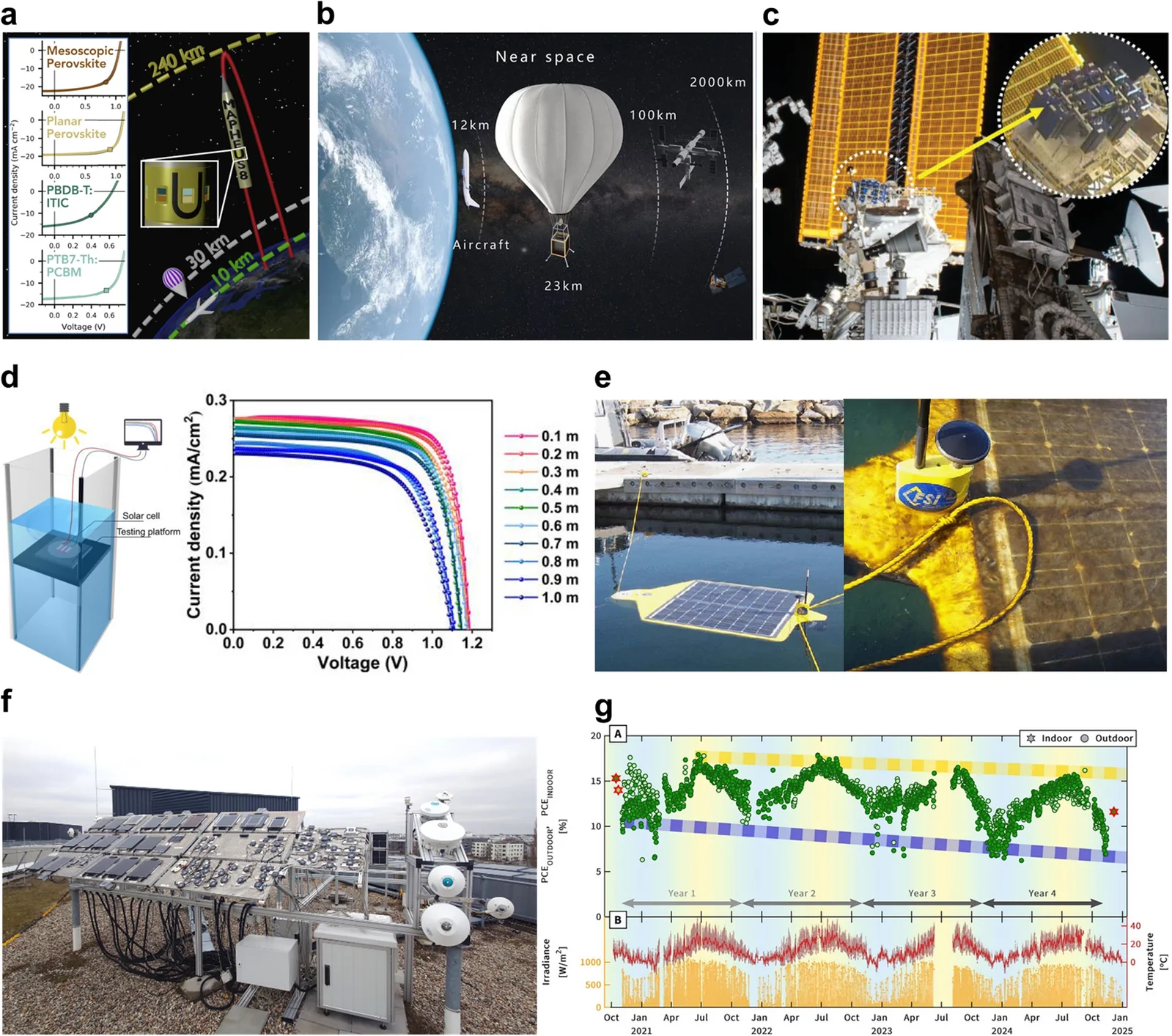
PSCs Under Real Extreme Conditions **a** Schematic overview of the MAPHEUS-8 sounding rocket flight [201]. Copyright 2020, Elsevier. **b** Schematic of a high-altitude balloon operating in near-space conditions [198]. Copyright 2022, John Wiley and Sons. **c** The International Space Station (ISS) on day 52 of the MISSE-13 mission; the yellow arrow indicates the location of the perovskite flight sample aboard the ISS [192]. Copyright 2023, John Wiley and Sons. **d** Schematic of underwater testing procedures for solar cells, along with *J*–*V* curves of CsPbIBr_2_ PSCs measured at various water depths [204]. Copyright 2023, American Chemical Society. **e** A solar-powered AUV covered in organic matter due to biofouling after 25 d of deployment [59]. Copyright 2023, Springer Nature. **f** Outdoor test field with PSCs; the area containing small-area devices is highlighted in green. Spectrometers used during the experiment are visible on the right [209]. Copyright 2025, John Wiley and Sons. **g** Long-term outdoor PCE, encapsulation backside device temperature, and in-plane irradiance for two 1 cm^2^ single-junction PSCs with identical architecture and encapsulation

In 2019, Tu et al. investigated the stability of mixed-cation PSCs under near-space conditions (~ 35 km altitude) and AM0 illumination [200]. They also assessed the effect of UV-filter coverage on device stability. A polyimide filter enhanced stability; with the filter, the device retained over 95% of its initial PCE (6.28%) for more than 1 h during near-space exposure. In 2020, Reb et al. conducted a spaceflight experiment using MAPbI_3_ PSCs onboard the MAPHEUS-8 sounding rocket (Esrange, Sweden), which reached an apogee of 239 km (Fig. 8a) [201]. Device performance was monitored over approximately 6 min, with cell temperatures ranging from 30 to 60 °C. Under direct solar irradiation, the PSCs delivered power densities exceeding 14 mW cm^−2^; under scattered-light conditions, they still produced 0.3–0.8 mW cm^−2^, with *V*_OC_ ≈ 0.85 V and FF > 60%, indicating suitability for deep-space-like low-irradiance environments. Although the presence of a window and oblique illumination precludes direct comparison with ground-based measurements, the results align with AM0 expectations and demonstrate favorable specific power, reinforcing the potential of PSCs for space applications.

Subsequent studies extended the test duration of PSCs in space-relevant conditions. In 2022, Wang et al. reported in situ performance monitoring of PSCs for 19 h in near space (~ 24 km altitude), providing the first diurnal performance profile (Fig. 8b) [198]. The devices maintained essentially constant *V*_OC_ and FF regardless of fluctuations in ambient temperature and irradiance. The integrated energy output reached 0.0869 kWh cm^−2^, with a maximum MPP of 11.9 mW cm^−2^ at solar noon, underscoring the viability of PSCs for sustained operation in near-space environments. In 2023, Delmas et al. reported a 10-month LEO exposure of a MAPI3 perovskite film deposited on borosilicate glass, protected by a 50 nm SiO_2_ barrier, and encapsulated with the space-grade silicone DC 93-500 [192]. The sample was mounted on the International Space Station in a zenith-facing (Sun-exposed) position from March 2020 to January 2021 (Fig. 8c). No irreversible radiation damage was observed, demonstrating that an appropriate combination of barrier layers and encapsulation can enable perovskite materials to endure long-term LEO exposure. Furthermore, during 15 h of post-flight light soaking, no photodarkening or defect-induced spectral broadening occurred; instead, photohealing was observed, evidenced by a reduction in surface defects. These findings highlight the resilience of perovskite materials under extended space conditions and confirm that space-induced stressors can be effectively mitigated, in agreement with theoretical predictions.

These real-environment studies underscore the promising applicability of PSCs in space and high-altitude conditions; however, additional launches and in-orbit evaluations remain necessary for rigorous validation. Moreover, the individual effects of rapid thermal cycling, radiation, and vacuum are not yet fully resolved. Ongoing efforts should aim to disentangle these stressors, enabling the development of more robust design rules for reliable space deployment.

### Underwater

Early studies established the spectral and attenuation limitations of underwater photovoltaics. While the optimal single-junction absorber bandgap at the terrestrial surface lies between approximately 1.2 and 1.4 eV, the attenuation of near-infrared light in water shifts this optimum to higher values, ~ 1.8–2.1 eV at ~ 4 m depth and ~ 2.4 eV at ~ 50 m [59, 202]. Wide-bandgap solar cells, therefore, show strong potential for efficient underwater operation. In this context, perovskites offer a distinct advantage due to their readily tunable bandgap via halide composition. For example, FAPbI_3_ exhibits a bandgap of ~ 1.5 eV, while FAPbBr_3_ reaches ~ 2.3 eV, with intermediate values achievable through iodide–bromide mixing. Accordingly, several studies have employed Br-rich perovskites to evaluate PSC performance in underwater environments.

Shao et al. addressed underwater applicability by selecting a wide-bandgap FAPbBr_3_ absorber, whose spectral response aligns well with the green-dominated underwater spectrum [203]. They minimized interfacial losses by introducing a simple self-assembled monolayer on the perovskite surface, increasing the AM1.5G PCE from 6.44 to 7.49%. To assess relevance for shallow-water conditions, the devices were further tested under monochromatic 520 nm illumination at low irradiance (4.8 mW cm^−2^), achieving a PCE of 30.0%, consistent with enhanced spectral matching and reduced carrier recombination in underwater settings. Wang et al. evaluated the underwater suitability of CsPbIBr_2_ (bandgap ~ 2.1 eV) [204]. Given the susceptibility of mixed-halide perovskites to light-induced halide migration and subsequent domain segregation into I-rich and Br-rich phases, which degrades device performance, they employed a Zn(C_6_F_5_)_2_ additive to suppress halide migration. The resulting devices exhibited *V*_OC_ = 1.41 V and PCE = 12.57% under AM1.5G illumination, with improved operational stability. Under submerged conditions at ~ 1 m depth, the devices achieved a PCE of 14.18% with an output power density of 0.162 mW cm^−2^, highlighting the potential of CsPbIBr_2_ for shallow-water photovoltaic applications (Fig. 8d).

To address the fundamental challenge of water ingress in underwater applications, advanced encapsulation strategies have been developed. These include poly(isobutylene) (PIB)-based edge-sealed barriers with ultralow water vapor transmission rates (WVTRs), inorganic/organic multilayer laminates, and corrosion-resistant electrodes and transport layers qualified through salt-mist and immersion testing protocols [171, 205, 206]. Such encapsulation systems not only resist water permeation but also suppress Pb leaching, with studies demonstrating maintained device performance even under saline conditions. For instance, Barichello et al. reported PIB-encapsulated FAPbBr_3_ PSCs operating in 3.5 wt% NaCl saline solution [171]. After 10 days of immersion, Pb release remained within stringent drinking-water safety limits.

Although biofouling has not yet been extensively studied in the context of underwater PSCs, it is a critical consideration for long-term deployment, as illustrated in Fig. 8e [59, 207]. Mitigation strategies include the application of chemically active biocide-based paints, non-adhesive surfaces, and fouling-release coatings. To preserve optical transparency, advanced coating technologies such as ClearSignal—a non-toxic, visible-light-transparent antifouling treatment developed by Severn Marine Technologies and Mid Mountain Materials—may be suitable [208]. ClearSignal has already been used on non-solar-powered AUVs during transatlantic missions, where, after a 7-month voyage covering over 4600 miles, the coated hull sections showed minimal to no biofouling.

For the practical deployment of PSCs in underwater environments, further research is required to ensure the stability and efficiency of wide-bandgap absorbers, to develop advanced encapsulation strategies capable of withstanding diverse aquatic conditions, and to mitigate biofouling. These efforts are essential to establishing design principles that enable the transition of PSCs from laboratory-scale demonstrations to reliable underwater photovoltaic technologies.

### Terrestrial Extreme Environments

Although PSCs have not yet been systematically tested in desert or polar regions, several outdoor studies offer valuable insights into device behavior under real-world terrestrial conditions across varying seasons [91, 209–212]. These evaluations capture the combined effects of temperature fluctuations, irradiance variation, and spectral shifts, which resemble key aspects of extreme terrestrial climates.

Remec et al. conducted a four-year outdoor study (2021–2025) in Berlin, Germany, on encapsulated PSCs to investigate seasonal impacts on device performance (Fig. 8f) [209]. The operating temperature ranged from approximately 0 °C in winter to 40 °C in summer. The results demonstrated promising long-term stability: Peak summer PCE values remained largely unchanged over the first two summers, with only an absolute decline of ≈2% observed between the first and fourth summers. However, the devices exhibited pronounced seasonality, with winter performance up to 30% lower than peak summer values (Fig. 8g). The reduced winter performance was primarily attributed to metastability arising from unsaturated light soaking under cold, low-irradiance conditions. Addressing this metastability will be essential for advancing PSCs toward commercial viability in temperate and extreme terrestrial environments.

Fei et al. fabricated encapsulated perovskite mini-modules (17.88 cm^2^) and conducted outdoor stability testing in Colorado, USA [91]. This study incorporated a strong-bonding HTL specifically designed to mitigate UV-induced degradation of the perovskite absorber. During testing, the module surface temperature reached 50 °C. The best-performing mini-module sustained a peak operating PCE of 17.5% after 10 weeks, while the average PCE across all mini-modules remained above 16%. Notably, the daily PCE of the top device, installed in September 2023, remained above 16% for 29 weeks, demonstrating robust module-level stability enabled by chemical interface engineering.

Dust accumulation on the glass covers of PV systems gradually reduces transmittance and, consequently, energy conversion efficiency [213]. Studies conducted in desert regions of China, India, and the Arabian Peninsula have shown that dust deposition can reduce the energy yield of Si-based PV systems by up to 25% [214]. To address this issue, self-cleaning films can be integrated with PSCs. For instance, Shirazi et al. developed a bioinspired self-cleaning film to enhance both the operational lifetime and efficiency of PSCs [215]. Mimicking the hierarchical surface structure of leek leaves, they fabricated a cellulose-based multifunctional coating that exhibits strong anisotropic light scattering and superhydrophobicity. With an additional carnauba wax layer, the film achieved an anisotropic contact angle of 160°, enabling water repellency that facilitates self-cleaning and reduces dust accumulation, thereby maintaining high transmittance and maximizing light absorption over time. These findings underscore that, beyond intrinsic material stability, envelope technologies for optical management and surface protection are critical to ensuring long-term outdoor durability and commercial viability of PSCs. We summarize representative results of PSC operation under extreme conditions in Table 7.

**Table 7:** Representative results of PSC operation under extreme conditions

Environment	Conditions	Perovskite composition	Key focus	Results	References
Near-space	~ 32 km, ~ 5 h (> 3 h in the stratosphere)	MAPbI_3_	Real-flight validation of PSC operation under near-space stressors	PCE decreased from 14.6% to 9.3% (no complete failure during flight)	[199]
Near-space	~ 35 km, AM0 illumination	FA_0.9_Cs_0.1_PbI_3_	Polyimide UV-filter coverage	> 95% of initial PCE (6.28%) retained for > 1 h	[200]
Space	Apogee 239 km, ~ 6 min monitoring, cell temperature 30–60 °C	MAPbI_3_	In-flight performance validation under space-relevant irradiance	Power density > 14 mW cm^−2^ (Direct sunlight)	[201]
Near-space	~ 24 km, 19-h in situ monitoring	Cs_0.05_MA_0.15_FA_0.80_Pb(I_0.85_Br_0.15_)_3_	Long-duration in situ monitoring (diurnal profile) under near-space conditions	*V*_OC_ and FF essentially constant, maximum MPP 11.9 mW cm^−2^ at solar noon	[198]
Space	International Space Station, 10 months	MAPbI_3_	SiO_2_ barrier + space-grade silicone encapsulation	No irreversible radiation damage	[192]
Underwater	520 nm monochromatic light at 4.8 mW cm^−2^	FAPbBr_3_	SAM on perovskite surface	AM1.5G PCE: 6.44% → 7.49%; under 520 nm low irradiance: PCE 30.0%	[203]
Underwater	~ 1 m depth submerged conditions	CsPbIBr_2_	Zn(C_6_F_5_)_2_ additive	AM1.5G: PCE 12.57%; submerged: PCE 14.18%	[204]
Underwater	3.5 wt% NaCl solution, 10 days	FAPbBr_3_	PIB-based edge-seal encapsulation	Pb release remained within stringent drinking-water safety limits after 10 days	[171]
Terrestrial	4 years, operating temperature ~ 0 °C (winter) to ~ 40 °C (summer)	Cs_0.15_FA_0.85_PbI_2.55_Br_0.45_	Robust module-scale encapsulation	Peak summer PCE largely unchanged over first two summers	[209]
Terrestrial	Device surface temperature up to 50 °C,	FA_0.9_Cs_0.1_PbI_3_	Strong-bonding HTL	Mini-module maintained PCE > 16% for 29 weeks	[91]

## Conclusion and Outlook

Over the past decade, PSCs have achieved remarkable progress in both efficiency and stability. These advancements stem from improvements in device architecture, the use of additives, surface and grain-boundary passivation strategies, and CTL engineering. Notably, several studies have demonstrated multiyear operational stability in outdoor conditions, indicating that PSCs are approaching the performance thresholds required for commercialization in temperate terrestrial environments.

**Fig. 9:**
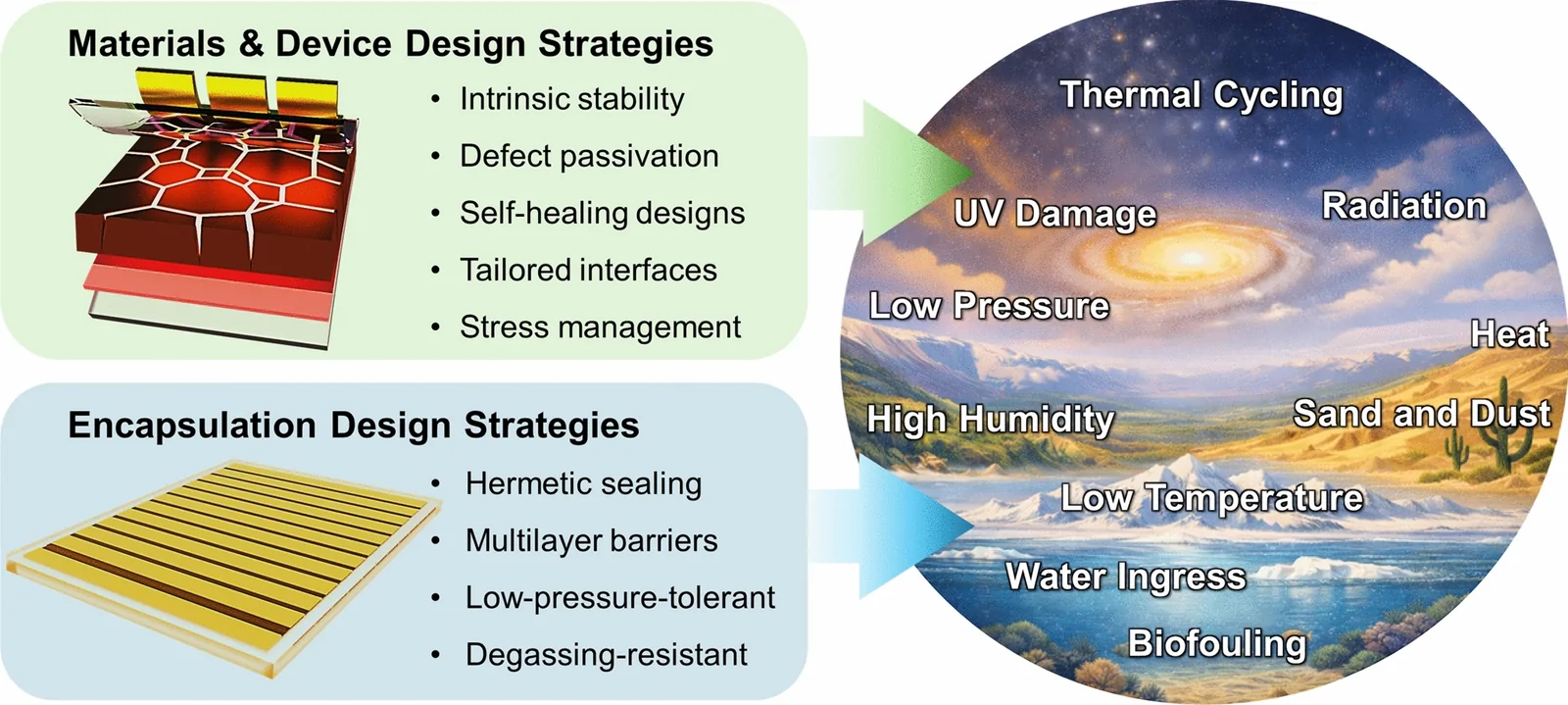
Schematic overview of key challenges to be overcome for the reliable deployment of PSCs in extreme environments

The next major challenge lies in extending the applicability of PSCs to extreme environments on Earth and in space, where harsh stressors such as intense illumination, thermal cycling, and humidity can accelerate degradation, owing to the intrinsically fragile nature of halide perovskites (Fig. 9). Although substantial effort is still required, emerging strategies—including advanced encapsulation, defect passivation, and self-healing approaches—provide promising pathways for overcoming these limitations.

From a materials standpoint, we propose several research directions to enhance the viability of PSCs in extreme environmental applications:

1. *Enhancing intrinsic stability of perovskites*: While various strategies have been proposed to mitigate degradation under external stressors, rigorous testing is still necessary to determine whether these materials can sustain performance under harsh illumination, elevated temperatures, and high humidity. Inorganic-based perovskites are generally expected to outperform their organic counterparts under such conditions. For instance, bilayer structures incorporating inorganic perovskites in place of conventional organic passivation agents have demonstrated potential [216]. Additionally, all-inorganic perovskites, low-dimensional perovskite structures, and double perovskite compositions are anticipated to offer improved compatibility with extreme environments [216–219]. However, systematic development in these material classes remains limited, as most extreme-environment testing to date has focused on hybrid organic–inorganic perovskites. Therefore, comprehensive comparative studies, rational material selection, and targeted optimization are essential to advancing the applicability of these emerging perovskite systems in extreme conditions.
2. *Exploiting self-healing properties*: Halide perovskites exhibit an intrinsic capacity for partial self-healing, as some degradation reactions induced by light, heat, and moisture are at least partially reversible [31, 56, 117, 148]. However, complete recovery is rarely achieved, since these degradation pathways often produce volatile by-products, such as MA, FA, or HI, that readily escape from the film. The loss of these components results in irreversible changes to the stoichiometry and crystallinity, ultimately compromising long-term device stability. To fully exploit the self-healing potential of halide perovskites, future materials design should focus on suppressing irreversible degradation mechanisms while enabling regulation or retention of volatile species. Strategies may include incorporating host matrices or molecular scaffolds that trap volatile compounds near the perovskite crystals, allowing for their reintegration during recovery. Alternatively, chemical modifications that reduce the volatility of decomposition products or stabilize labile bonds could extend the window of reversibility and improve recovery efficiency.
3. *Advancing encapsulation technologies*: Encapsulation provides the primary defense against environmental degradation; however, the development of specialized encapsulation materials for PSCs remains relatively underexplored. Future research should prioritize the design of encapsulants that are chemically inert with respect to perovskite materials, impermeable to small-molecule penetrants, and mechanically robust under repeated thermal and mechanical stress [187]. Hybrid encapsulation schemes—such as those combining rigid inorganic barriers with flexible organic coatings, multilayer polymer–glass laminates, or nanocomposite films—may offer a suitable balance between hermetic sealing and mechanical compliance. Moreover, encapsulation performance must be assessed not only under standard damp-heat protocols but also under comprehensive multistress conditions that simulate real-world environments, including simultaneous exposure to heat, humidity, light, electrical bias, and radiation. For aerospace and high-altitude applications, the development of low-pressure-tolerant encapsulants and degassing-resistant architectures will be particularly critical.

Beyond materials development, we propose several broader guidelines:

1. *Increased real-world exposure*: To date, most stability assessments of PSCs have been conducted under controlled laboratory conditions. While these tests yield valuable mechanistic insights, they often fail to capture the complexity of extreme real-world environments, which impose dynamic combinations of stressors that are difficult to replicate in isolation. To validate laboratory findings and uncover degradation pathways that manifest only under authentic environmental operation, more extensive deployment and monitoring of PSCs in genuine field conditions, such as long-duration outdoor tests, balloon flights, or space exposure missions, are urgently needed.
2. *Standardization of extreme-environment testing protocols*: Current PSC stability assessments primarily rely on accelerated lifetime testing protocols, such as the IEC 61215 standard, which emphasize damp-heat exposure, thermal cycling, and light soaking [220]. However, these protocols were originally developed for Si photovoltaics and do not adequately capture degradation mechanisms specific to perovskite materials. Moreover, extreme environments introduce coupled stressors—including rapid thermal cycling, vacuum, high-energy radiation, and corrosive chemical species—that are not addressed in existing qualification schemes. To enable reliable deployment of PSCs beyond mild terrestrial climates, standardized protocols must be developed to reflect these multistress conditions. These frameworks should incorporate combined stress testing (heat + humidity + light + bias + radiation), realistic thermal-cycling ranges, and vacuum or low-pressure conditions. Additionally, they should emphasize reproducibility, interlaboratory comparability, and statistically significant device populations.

While a specific qualification protocol for PSCs in extreme environments has not yet been established, they can be evaluated using reference frameworks developed for conventional photovoltaic technologies operating under similar conditions. For space applications, qualification typically follows dedicated space solar array standards such as AIAA S-111A, which address tolerance to high-energy radiation, vacuum exposure, and wide operating temperature ranges [33]. Underwater and marine operation is more appropriately assessed using ingress and corrosion protocols defined in IEC 60068 (e.g., water immersion and salt-mist tests), where encapsulation integrity and long-term environmental isolation critically govern device stability [221, 222]. Desert environments are commonly evaluated using terrestrial PV qualification standards, particularly IEC 61215, including damp-heat testing as a worst-case thermal–moisture stress, supplemented by sand and dust abrasion (IEC 60068-2-68) and accelerated UV aging protocols (ASTM G154) [223–225]. For polar operation, low-temperature durability and mechanical reliability are typically assessed using thermal cycling and mechanical load tests defined in IEC 61215, together with temperature-change protocols (IEC 60068-2-14) and snow/ice load considerations (ISO 12494) [223, 226, 227]. High-altitude operation, reflecting an intermediate regime between terrestrial and space environments, is generally evaluated using low-pressure and thermal-shock tests specified in IEC 60068-2-13 and IEC 60068-2-14 [226, 228]. Collectively, these reference protocols provide a practical foundation for developing PSC-specific extreme-environment testing criteria that are tailored to the dominant stressors of each application.

3. *Understanding coupled stressors*: Most reported studies rely on single-factor stress testing, in which light, heat, or humidity is applied independently. However, in real-world environments, these stressors act simultaneously and interact through synergistic or competitive mechanisms. For example, illumination combined with oxygen accelerates photooxidative reactions; humidity under bias stress promotes ion migration and interfacial instability; and thermal cycling in the presence of moisture exacerbates mechanical fatigue and delamination [88, 139]. Capturing such effects necessitates multistress testing protocols that more accurately reflect actual operating conditions, supported by advanced in situ characterization tools capable of resolving dynamic degradation processes. In this context, artificial intelligence (AI) can complement experimental studies by integrating heterogeneous datasets, constructing physics-informed surrogate models, and enabling rapid, less biased decision-making [229].

4. *Indoor Light Recycling for Enclosed Platforms*: In enclosed platforms such as space stations and underwater habitats, access to direct solar irradiance is often intermittent or absent. Under these conditions, indoor light recycling can provide a route to harvest scattered and reflected photons from artificial interior lighting, thereby reducing net power demand and enabling maintenance-free powering of distributed sensors and other low-power electronics. PSCs are particularly suitable for this application because they can maintain high PCE at low irradiance and have bandgaps that can be tailored to match indoor illumination spectra [230]. Accordingly, integrating perovskite photovoltaics into interior surfaces, together with packaging that meets fire-safety, low-outgassing, and long-term reliability requirements, could be a promising direction for extreme-environment energy systems.

5. *Perovskite-Based Tandems for Extreme Environments*: Tandem photovoltaics can increase areal power output, which is particularly relevant for space deployment where the available area is limited. In this context, perovskite-based tandems, including perovskite/silicon, all-perovskite, and perovskite/Cu(In,Ga)Se_2_ (CIGS) architectures, are promising for extreme environments. If the efficiency gain exceeds the associated mass penalty, tandem architectures can also improve specific power. Considering that space solar arrays typically operate at around 30 W kg^−1^ at the system level [231], reports of ultrathin flexible perovskite devices exhibiting device-level specific powers on the order of tens of kW kg^−1^ (e.g., >20 kW kg^−1^) imply that perovskite-based tandem architectures could further increase specific power while maintaining lightweight implementations [232, 233].

Nevertheless, for extreme-environment deployment, the efficiency benefit must be supported by reliable operation under radiation and thermomechanical stress. In a prior study under 68 MeV proton irradiation, monolithic perovskite/silicon heterojunction tandems exhibited a rapid loss of performance, decreasing to approximately 1% of the initial PCE after a fluence of about 10^11^ p^+^ cm^−2^, whereas monolithic perovskite/CIGS tandems retained more than 85% of the initial PCE even at 2 × 10^12^ p^+^ cm^−2^ [234]. These results indicate that radiation-tolerant configurations, such as all-perovskite and perovskite/CIGS tandems, may be preferred over silicon-based tandems for space applications. At the same time, the increased number of interfaces in tandem stacks can exacerbate delamination and cracking under thermal cycling due to thermal expansion mismatch. Therefore, perovskite tandems for extreme environments should be advanced through concurrent optimization of device architecture and packaging, including CTE matching, stress-buffer layers, and ultrathin encapsulation, to achieve both high specific power and long-term operational reliability.
